# Subcomponent Self‐Assembly of a Cyclic Tetranuclear Fe^II^ Helicate in a Highly Diastereoselective Self‐Sorting Manner

**DOI:** 10.1002/chem.201903164

**Published:** 2019-08-28

**Authors:** Jana Anhäuser, Rakesh Puttreddy, Lukas Glanz, Andreas Schneider, Marianne Engeser, Kari Rissanen, Arne Lützen

**Affiliations:** ^1^ Kekulé-Institut für Organische Chemie und Biochemie Rheinische Friedrich-Wilhelms-Universität Bonn Gerhard-Domagk-Strasse1 53121 Bonn Germany; ^2^ Department of Chemistry University of Jyväskylä P.O. Box 35 40014 Jyväskylä Finland

**Keywords:** chiral self-sorting, cyclic helicates, paracyclophanes, self-assembly, supramolecular chemistry

## Abstract

An enantiomerically pure diamine based on the 4,15‐difunctionalized [2.2]paracyclophane scaffold and 2‐formylpyridine self‐assemble into an optically pure cyclic metallosupramolecular Fe_4_L_6_ helicate upon mixing with iron(II) ions in a diastereoselective subcomponent self‐assembly process. The cyclic assembly results from steric strain that prevents the formation of a smaller linear dinuclear triple‐stranded helicate, and hence, leads to the larger strain‐free assembly that fulfils the maximum occupancy rule. Interestingly, use of the racemic diamine also leads to a racemic mixture of the homochiral cyclic helicates as the major product in a highly diastereoselective narcissistic chiral self‐sorting manner given the fact that the assembly contains ten stereogenic elements, which can in principle give rise to 149 different diastereomers. The metallosupramolecular aggregates could be characterized by NMR, UV/Vis and CD spectroscopy, mass spectrometry, and X‐ray crystallography.

## Graphical abstract

**Chiral self‐sorting**: Only one out of 149 possible diastereomeric cyclic tetranuclear helicates forms largely preferred in an approximately 460‐times higher yield than expected for a purely statistical process upon highly diastereoselective subcomponent self‐assembly of a mixture of a pseudo *meta* difunctionalized [2.2]paracyclophane scaffold, 2‐formyl‐pyridine and iron(II) triflate hexahydrate in a highly selective narcissistic non‐integrative chiral self‐sorting manner (see scheme).

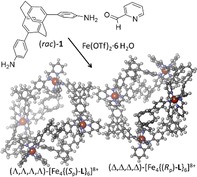

A prominent feature of most natural self‐assembled (supra‐) molecular architectures is chirality, which also has a marked influence on their structural and functional properties. Therefore, a lot of efforts were made to achieve high‐fidelity stereoselectivity in self‐assembly processes leading to stereochemically well‐defined aggregates in supramolecular chemistry in general^1^ and in metallosupramolecular chemistry in particular.^2^ In this respect, helicates^3^ have proven to be versatile model systems as they are by itself chiral objects and the mechanical coupling of two or more metal binding sites in a ligand strand offers an outstanding opportunity to control the relative stereochemistry of stereogenic metal centres, which is usually difficult to achieve otherwise.^4^ As was already nicely shown by Lehn and co‐workers in 1991 when they reported the diastereoselective self‐assembly of an enantiomerically pure tris(bipyridine) ligand into enantiomerically pure double‐stranded trinuclear linear helicates upon coordination to silver(I) or copper(I) ions,^5^ this approach usually gives rise to excellent stereoselectivity if the ligands are properly designed in a way that the formation of the helicate does not impose too much steric strain on the assembly, which could overcompensate for the energetical differences between the possible stereoisomers.3p However, the synthetic effort to access such well behaving ligands is usually quite substantial, and hence, this approach has mainly been restricted to di‐ and trinuclear double‐ or triple‐stranded linear helicates to date. This is quite an achievement in view of the fact, that the self‐assembly of enantiomerically pure or racemic ligands to a trinuclear triple‐stranded helicate can, in principle, result in the formation of six or twelve different diastereomers, respectively. Nevertheless, we were wondering if we could push this approach even further.

Thus, cyclic helicates3s caught our attention because they can be composed of much more components, and therefore, can give rise to much more complex mixtures of diastereomers. Despite the fact that the first example was already reported in 1996 by J.‐M. Lehn,^6^ cyclic helicates are still rather rare, because the outcome of the self‐assembly processes cannot be predicted as well as for their linear analogues in terms of both aggregate composition and stereoselectivity.^7^ The most promising design strategies involve the use of anion template effects,^6^, 7c, 8, 9, 10, 11 control by metal‐ion radii^12^ or additional ligand–ligand interaction,^13^ or the implementation of steric strain that prevents the formation of linear helicates.^14^, ^15^, ^16^, ^17^, ^18^ In fact, there are only two studies up to now that involve enantiomerically pure bis(chelating) ligands that could be demonstrated to undergo diastereoselective self‐assembly to enantiomerically pure cyclic helicates.13c, 14 In both of these, the individual metal centres are bridged by single ligand strands, which have stereogenic elements in their outer periphery. However, neither have ligand strands bearing a stereogenic element in between the two‐metal binding site ever been successful in this sense, nor have racemic ligands ever been tested with regard to their chiral self‐sorting behaviour in this context.

Our design of a suitable ligand structure to explore this is based on the following hypotheses: the ligand should be dissymmetric, curved, and rather rigid to ensure diastereoselectivity. Furthermore, it should be sterically congested to a certain degree so that only two ligands could bridge two adjacent metal centres, and thus, preventing formation of a dinuclear triple‐stranded helicate upon coordination to metal ions preferring an octahedral coordination by three chelating ligands, for example, iron(II) ions. In such a situation, the metal ions’ coordination spheres can only be completed in meridional configurations. In such a scenario, the curved shape of the ligands and the tendency to fulfil the maximum occupancy rule should ensure formation of the smallest cyclic helicate that does not suffer from too much steric strain, in which adjacent metal centres are alternately bridged by one and two ligand strands similar to the octanuclear cyclic helicate presented by Ward and co‐workers.^9^

According to these criteria, we envisioned a subcomponent self‐assembly approach^19^ starting from diamine **1** based on the rigid V‐shaped scaffold of a pseudo‐*meta* difunctionalized [2.2]paracyclophane (opening angle of 120° ±12°). Compound (*rac*)‐**1** could be prepared in two steps from readily accessible (*rac*)‐4,15‐diodo[2.2]paracyclophane via a Suzuki cross‐coupling and subsequent deprotection (Scheme 1; see the Supporting Information for details).

**Scheme 1:**
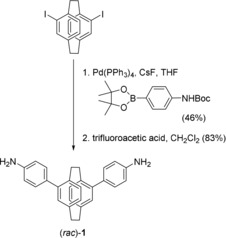
Synthetic route to planar chiral ligand precursor (*rac*)‐**1**.

For the resolution of racemic diamine (*rac*)‐**1**, we drew on our experience to resolve planar chiral [2.2]paracyclophanes by HPLC on a chiral stationary phase.^20^ Separation could be achieved on analytical, as well as on semi‐preparative scale, on a CHIRALPAK^®^ IB column (see the Supporting Information) to get both enantiomers of **1**. Good‐quality crystals of second eluting (+)‐**1** were obtained by slow evaporation of a solution in methanol with the addition of one drop of trimethylamine and XRD analysis confirmed the (*S*_p_)‐configuration of this enantiomer (see the Supporting Information for a picture and further details of the structure).^21^

To explore the subcomponent self‐assembly processes using enantiomerically pure diamines **1**, a 3:6:2 molar ratio of (*R*_p_)/(*S*_p_)‐**1**, 2‐formylpyridine, and iron(II) triflate hexahydrate in acetonitrile was heated at 70 °C for 15 hours. The desired metallosupramolecular aggregates were isolated as purple coloured solids by adding diethyl ether into corresponding acetonitrile reaction mixtures (for more details, see the Supporting Information). The ESI(+) mass spectra of solutions of these complexes in acetonitrile clearly revealed the formation of the desired tetranuclear [Fe_4_(**L**)_6_](OTf)_8_ complexes containing ten stereogenic elements (see the Supporting Information) with **L** being the bis(pyridylimine) ligand obtained from the condensation of **1** with 2‐formylpyridine (Figure 2).

Single crystals of [Fe_4_{(*R*_p_)‐**L**}_6_](OTf)_8_ and [Fe_4_{(*S*_p_)‐**L**}_6_](OTf)_8_ suitable for X‐ray diffraction analysis were obtained by slow diffusion of *tert*‐butyl methyl ether into the respective acetonitrile solutions. These two isostructural Fe^II^ complexes crystallize in the orthorhombic chiral space group *P*2_1_2_1_2_1_ and are isomorphic. Their asymmetric unit contains six crystallographically different L molecules that are arranged in an in‐and‐out fashion around four Fe^II^ centres, which defines the topology of a circular helicate as shown in Figure 1 (for details on [Fe_4_{(*S*_p_)‐**L**}_6_](OTf)_8_, see the Supporting Information). Each of the Fe^II^ ions in the tetranuclear assemblies is coordinated in an octahedral environment and each complex exhibits either only (Δ)‐ or (Λ)‐configurated metal centres in (Δ,Δ,Δ,Δ)‐[Fe_4_{(*R*_p_)‐**L**}_6_](OTf)_8_ and (Λ,Λ,Λ,Λ)‐[Fe_4_{(*S*_p_)‐**L**}_6_](OTf)_8_, respectively.

**Figure 1:**
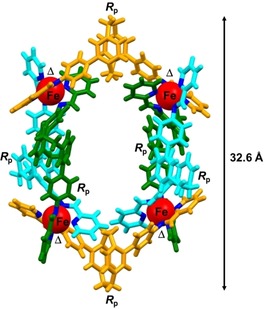
X‐ray crystal structure of homochiral metallosupramolecular cyclic helicate (Δ,Δ,Δ,Δ)‐[Fe_4_{(*R*_p_)‐**L**}_6_](OTf)_8_ in capped stick model. Counterions and solvent molecules were omitted for clarity.

The diastereoselectivity of the self‐assembly processes was further confirmed by NMR spectroscopy. Analysis of the ^1^H and the ^1^H 2D DOSY NMR spectra revealed single set of signals, symmetry and chemical shifts of which are in accordance with the M_4_L_6_ assemblies observed in the X‐ray crystal structures (Figure 2 b and the Supporting Information).

**Figure 2:**
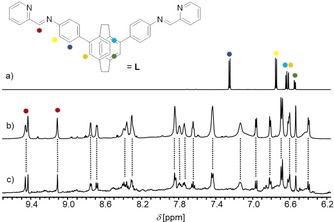
Aromatic region of the ^1^H NMR spectra (700.4 MHz, [D_3_]acetonitrile, 298 K) of a) diamine **1**, b) homochiral cyclic helicate (Δ,Δ,Δ,Δ)‐[Fe_4_{(*R*_p_)‐**L**}_6_](OTf)_8_, and c) the outcome of the subcomponent self‐assembly of (*rac*)‐**1**, 2‐formylpyridine and Fe^II^ triflate hexahydrate.

Furthermore, the enantiomerically pure helicates could also be characterized by UV/Vis and CD spectroscopy—the latter giving rise to mirror‐image spectra as was expected (see the Supporting Information). Thus, we can conclude that the self‐assembly of the enantiomerically pure diamines (*R*_p_)‐**1** and (*S*_p_)‐**1** occurs in a completely diastereoselective manner leading to only one of the seven possible diastereomers, which corresponds to a 16 times higher yield than expected for a purely statistical assembly of all possible diastereomers (see the Supporting Information).

However, the situation gets much more complex if the subcomponent self‐assembly process starts from (*rac*)‐**1**, and hence, we explored this next to elucidate its (diastereo‐)selectivity and possible chiral self‐sorting effects. First, we checked the composition of the resulting aggregates by ESI(+) MS experiments again confirming that tetranuclear complexes are formed as the major species from the racemic material. But in this case, they are also accompanied by a small portion of dinuclear complexes (see the Supporting Information).

The ^1^H NMR spectra of enantiomerically pure (Δ,Δ,Δ,Δ)‐[Fe_4_{(*R*_p_)‐**L**}_6_](OTf)_8_ (and (Λ,Λ,Λ,Λ)‐[Fe_4_{(*S*_p_)‐**L**}_6_](OTf)_8_) are almost identical to the spectrum obtained after mixing a 3:6:2 molar ratio of (*rac*)‐**1**, 2‐formylpyridine and iron(II) triflate hexahydrate (Figure 2 c). However, the ^1^H NMR spectrum recorded from the assemblies of the racemic starting material further accounts for less than 10 % of by‐products potentially due to formation of smaller dinuclear aggregates and/or minor amounts of tetranuclear diastereoisomers, thus translating into a relative yield of 90 % of the racemic mixture of homochiral stereoisomers (Δ,Δ,Δ,Δ)‐[Fe_4_{(*R*_p_)‐**L**}_6_](OTf)_8_ and (Λ,Λ,Λ,Λ)‐[Fe_4_{(*S*_p_)‐**L**}_6_](OTf)_8_. This was further corroborated by ^1^H‐2D‐DOSY NMR experiments and X‐ray crystallography. The size derived from DOSY NMR signals of the major product obtained from (*rac*)‐**1** is in good agreement with the values obtained for the homochiral enantiomers (r_H_=1.4 nm). Note, that the size of both the racemate and the enantiomerically pure M_4_L_6_ cyclic helicates confirmed by DOSY NMR experiments also agree with the solid‐state X‐ray crystal structures dimensions (see the Supporting Information for more details). The racemic tetranuclear complex crystallizes in the tetragonal centrosymmetric space group *I*4_1_/*acd*, suggesting that the unit cell contains a mixture of both enantiomers, (Δ,Δ,Δ,Δ)‐[Fe_4_{(*R*_p_)‐**L**}_6_](OTf)_8_ and (Λ,Λ,Λ,Λ)‐[Fe_4_{(*S*_p_)‐**L**}_6_](OTf)_8_ as shown in Figure 3.

**Figure 3:**
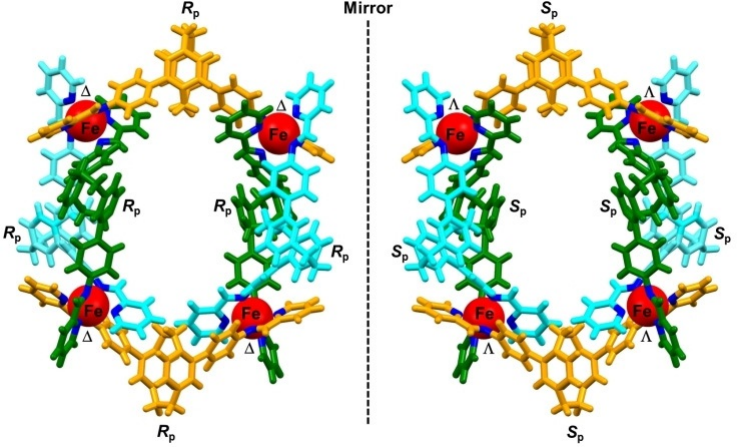
X‐ray crystal structure of racemic tetranuclear metallosupramolecular cyclic helicates, (Λ,Λ,Λ,Λ)‐[Fe_4_{(*S*_p_)‐**L**}_6_](OTf)_8_ and (Δ,Δ,Δ,Δ)‐[Fe_4_{(*R*_p_)‐**L**}_6_](OTf)_8_, depicted in isomeric forms using capped‐stick model. Counterions were omitted for clarity.

This behaviour is truly remarkable in view of the fact that the self‐assembly of (*rac*)‐**L** into a cyclic tetranuclear complex of this kind with ten stereogenic elements could, in principle, lead to 149 different diastereomers (see the Supporting Information).^22^ Hence, the fact that we observed the formation of a single one of these in more than 90 % yield instead of the <0.2 % expected if the process would proceed in a statistical manner clearly reveals the excellent diastereoselectivity of the self‐assembly process, which happens in a highly selective narcissistic non‐integrative chiral self‐sorting manner.^23^

In summary, we were able to synthesize a chiral diamine **1** based on a pseudo*‐meta* difunctionalized [2.2]paracyclophane scaffold and employ it in enantiomerically pure and racemic form to obtain tetranuclear cyclic iron(II) helicates upon subcomponent self‐assembly with 2‐formylpyridine and iron(II) triflate hexahydrate. The self‐assembly processes occurred in a highly diastereoselective manner leading to enantiomerically pure homochiral aggregates (Δ,Δ,Δ,Δ)‐[Fe_4_{(*R*_p_)‐**L**}_6_](OTf)_8_ and (Λ,Λ,Λ,Λ)‐[Fe_4_{(*S*_p_)‐**L**}_6_](OTf)_8_ when starting from enantiomerically pure (*R*_p_)‐**1** or (*S*_p_)‐**1**. However, much more remarkable: self‐assembly of (*rac*)‐**1** also leads to the racemic mixture of these homochiral complexes as the major product in an approximately 460 times higher yield than expected for a purely statistical assembly emphasizing the almost ideal preorganization of our ligand for the formation of this diastereomer in a highly selective narcissistic non‐integrative chiral self‐sorting manner. Hence, we were able to prove that oligonuclear circular helicates can indeed be prime examples how diastereoselectivity of self‐assembly processes can be pushed to new limits leading to aggregates with multiple stereogenic elements from components that contain only a single one if the ligand's components are designed in the proper way.

This shows nicely that even roughly 30 years after Lehn and co‐workers coined the term helicates,^24^ and the astonishing development this field took ever since, it still bears exciting new facets to be explored based on the achievements of the pioneers.

## Conflict of interest

The authors declare no conflict of interest.

## Supporting information

As a service to our authors and readers, this journal provides supporting information supplied by the authors. Such materials are peer reviewed and may be re‐organized for online delivery, but are not copy‐edited or typeset. Technical support issues arising from supporting information (other than missing files) should be addressed to the authors.

Supplementary
