# Supplementary material for: Subcomponent Self‐Assembly of a Cyclic Tetranuclear FeII Helicate in a Highly Diastereoselective Self‐Sorting Manner
Source: Chemistry. 2019 Aug 28;25(53):12294–7. doi: 10.1002/chem.201903164 (PMC6790559; doi:10.1002/chem.201903164)
Supplement: Supplementary file 1 — Supplementary [file CHEM-25-12294-s001.pdf]

CHEMISTRY
A European Journal

Supporting Information

Subcomponent Self-Assembly of a Cyclic Tetranuclear Fe^{II} Helicate in a Highly Diastereoselective Self-Sorting Manner

Jana Anhäuser,[a] Rakesh Puttreddy,[b] Lukas Glanz,[a] Andreas Schneider,[a]
Marianne Engeser,[a] Kari Rissanen,[b] and Arne Lützen*[a]

chem_201903164_sm_miscellaneous_information.pdf

Supporting Information
©Wiley-VCH 2016
69451 Weinheim, Germany

Abstract: An enantiomerically pure diamine based on the 4,15-difunctionalized [2.2]paracyclophane scaffold and 2-formylpyridine self-assemble into an optically pure cyclic metallosupramolecular Fe_4L_6 helicate upon mixing with iron(II) ions in a diastereoselective subcomponent self-assembly process. The cyclic assembly results from steric strain that prevents the formation of a smaller linear dinuclear triple-stranded helicate, and hence, leads to the larger strain-free assembly that fulfills the maximum occupancy rule. Interestingly, use of the racemic diamine also leads to a racemic mixture of the homochiral cyclic helicates as the major product in a highly diastereoselective narcissistic chiral self-sorting manner given the fact that the assembly contains ten stereogenic elements which can in principle give rise to 149 different diastereomers. The metallosupramolecular aggregates could be characterized by NMR, UV-Vis and CD spectroscopy, mass spectrometry and X-ray crystallography.

DOI: 10.1002/anie.2016XXXXX

Table of Contents

General experimental information 2
Synthetic Scheme 3
Synthesis and characterization of ligand precursors 4
Self-assembly and characterization of iron(II) complexes 12
Schematic lists of possible diastereomers of cyclic tetranuclear aggregates 21
X-ray crystallography 26

General experimental information

All reactions with air and moisture sensitive compounds were performed under argon atmosphere using standard Schlenk techniques, oven-dried glassware and dry solvents.

The following chemicals were obtained commercially from *Alfa Aesar*, *Sigma-Aldrich*, *Carbolution*, *Fluorochem* or *abcr* and used as received: *tert*-butyl-*N*-(4-(4,4,5,5-tetramethyl-1,3,2-dioxoborolan-2-yl)phenyl)carbamate, cesium fluoride, tetrakis(triphenylphosphine)-palladium(0), trifluoroacetic acid.

The following chemicals were synthesized according to known literature synthesis procedures: 4,15-diiodo[2.2]paracyclophane (*rac*)-**A**,[1] iron(II) triflate hexahydrate.[2]

All solvents were obtained from commercial sources. Dry solvents were obtained from the solvent purification system *MS-SPS 800* from *MBraun*. Other reaction solvents and solvents for specific rotation value, UV-Vis or CD measurements were solvents of *p.a.* grade. For flash column chromatography freshly destilled solvents of technical grade and for high performance liquid chromatography solvents of HPLC grade were used.

Thin-layer chromatography was performed on silica gel-coated aluminum plates with fluorescent indicator F254 from *Merck*. Detection was done by UV-light (254 and 366 nm).

Products were purified by flash column chromatography on silica gel 60 (particle size 0.040-0.063 mm) from *Merck* or on reversed phase silica gel (C18-RP, 17% C, 0.048-0.065 mm) from *Acros Organics*.

^{1}H, ^{13}C, H,H-COSY, HSQC, HMBC and ^{1}H-2D-DOSY NMR experiments were performed on a *Bruker Avance I* 500 spectrometer or a *Bruker Avance III HD* 700 spectrometer with a *cryo* probe. ^{1}H NMR chemical shifts are reported relative to residual non-deuterated solvent as internal standard. ^{13}C NMR chemical shifts are reported relative to deuterated solvent as internal standard. All shifts are reported on the δ scale in ppm and NMR multiplicities are abbreviated as s (singlet), d (doublet), t (triplet), dd (doublet of doublets), ddd (doublet of doublet of doublets) or m (multiplet). Coupling constants *J* are reported in Hertz. All spectra were processed using the *MestReNova 8.0.1* program from *Mestrelab*. ^{1}H-2D-DOSY NMR spectra were evaluated using the software *Topspin 3.5* from *Bruker* and the *Stokes-Einstein equation*, normally used for spherical particles, with a correction factor for ellipsoids (Equation S1).[3]

$$D = \frac{k_B\, T}{6\, \pi\, \eta f R_h}$$

Equation S1: *Stokes-Einstein equation*. D = diffusion constant, k_B = Boltzmann constant, T = temperature, η = viscosity of solvent, f = correction factor for ellipsoids, R_h = hydrodynamic radius.

(High-resolution) electrospray ionization mass spectra in positive mode (ESI(+)-MS) were measured on an *Orbitrap XL* mass spectrometer from *Thermo Fisher Scientific* or a *micrOTOF-Q* time-of-flight mass spectrometer from *Bruker Daltonik*.

Elementary analyses were performed on a *Vario EL* elemental analyzer from *Heraeus*.

Melting points were determined with a *DigiMelt MPA 161* instrument from *Stanford Research Systems*.

[1] G. Meyer-Eppler, R. Sure, A. Schneider, G. Schnakenburg, S. Grimme, A. Lützen, *J. Org. Chem.* **2014**, *79*, 6679-6687.
[2] K. S. Hagen, *Inorg. Chem.* **2000**, *39*, 5867-5869.
[3] a) K. E. Van Holde, *Physical Biochemistry*, Prentice-Hall, Englewood Cliffs, **1971**; b) A. Macchioni, G. Ciancaleoni, C. Zuccaccia, D. Zuccaccia, *Chem. Soc. Rev.*, 2008, **37**, 479-489.

UV-Vis spectra were measured on a *Specord 200* instrument from *Analytik Jena* and analyzed using the software *WinASPECT 1.7.2.0*. Quartz glass cuvettes from *Hellma Analytics* with a layer thickness of 10 mm (solutions of ligand precursors/ligands) and 0.01 mm (complex solutions) were used.

Circular dichroism (CD) spectra were measured on a *J-810* spectrometer from *Jasco*. Quartz glass cuvettes from *Hellma Analytics* with a layer thickness of 1 mm (solutions of ligand precursors/ligands) and 0.01 mm (complex solutions) were used.

Specific rotation values were measured on an *Anton Paar Model MCP 150* polarimeter with a standard wavelength of 589 nm using a cuvette with a layer thickness of 100 mm.

High performance liquid chromatography on analytical scale was performed on a *PLATINblue* HPLC system from *Knauer*, equipped with two pumps, an online degasser and a photodiode array detector *PDA-1* with a deuterium and tungsten-halogen lamp (190-1000 nm), and a *CHIRALPAK®* IB column (4.6 mm Ø, 250 mm) by *Daicel* was used. High performance liquid chromatography on semi-preparative scale was performed on an *Azura* HPLC system from *Knauer*, equipped with a binary HPG pump *P 6.1L*, an online degasser, a multi wavelength detector *MWL 2.1L* with deuterium lamp (190-700 nm) and a fraction collector, and a *CHIRALPAK®* IB column (20 mm Ø, 250 mm) by *Daicel* was used.

Synthetic Scheme

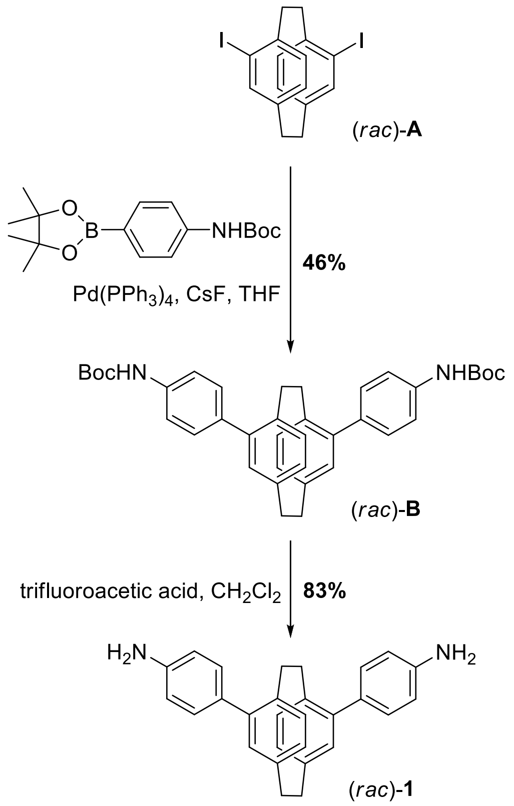

Scheme S1: Synthetic route to diamine (*rac*)-**1**.

Synthesis and characterization of ligand precursors

(*rac*)-4,15-Bis-((4,1-phenylene)-*N*-(*tert*-butyloxycarbonyl)amine)[2.2]paracyclophane ((*rac*)-B)

Under an atmosphere of argon, (*rac*)-**A**[1] (790 mg, 1.72 mmol, 1.00 eq.), *tert*-butyl-*N*-(4-(4,4,5,5-tetramethyl-1,3,2-dioxoborolan-2-yl)phenyl)carbamate (1.15 g, 3.61 mmol, 2.10 eq.), cesium fluoride (1.75 g, 10.3 mmol, 6.00 eq.), tetrakis(triphenylphosphine)palladium(0) (199 mg, 0.172 mmol, 10.0 mol%) were dissolved in dry tetrahydrofuran (80 mL). The reaction mixture was degassed at room temperature and then heated to reflux for 48 h. After cooling to room temperature the solution was quenched by the addition of saturated aqueous ethylenediaminetetraacetic acid disodium salt solution and dichloromethane. The mixture was extracted with dichloromethane. The combined organic layers were washed with brine, dried with anhydrous magnesium sulfate and the solvent was removed under reduced pressure. The crude product was subjected to flash column chromatography on silica gel (cyclohexane/ethyl acetate 10:1) and reversed phase silica gel (acetonitrile/water 3:2) to give (*rac*)-**B** (469 mg, 0.794 mmol, 46%) as a white solid.

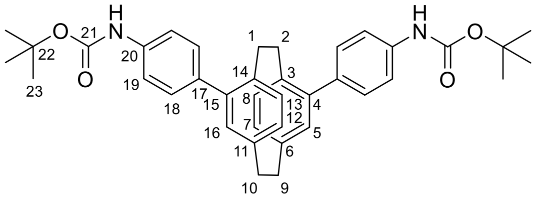

Chemical Formula: $C_{38}H_{42}N_2O_4$
Exact Mass: 590.3145 u
Molecular Weight: 590.76 gmol^{-1}

^{1}H NMR (499.1 MHz, $CDCl_3$, 298 K): δ[ppm] = 7.49-7.45 (m, 4 H, H-19), 7.44-7.40 (m, 4 H, H-18), 6.67 (d, 2 H, H-8, H-13, $^3J_{8,7}$ = $^3J_{13,12}$ = 7.8 Hz), 6.66 (d, 2 H, H-5, H-16, $^4J_{5,7}$ = $^4J_{16,12}$ = 1.9 Hz), 6.56 (s, 2 H, NH), 6.54 (dd, 2 H, H-7, H-12, $^3J_{7,8}$ = $^3J_{12,13}$ = 7.8 Hz, $^4J_{7,5}$ = $^4J_{12,16}$ = 1.9 Hz), 3.25-3.00 (m, 6 H, H-1, H-2, H-9, H-10), 2.52-2.42 (m, 2 H, H-1, H-2), 1.55 (s, 18 H, H-23).

^{13}C NMR (125.5 MHz, $CDCl_3$, 298 K): δ [ppm] = 153.0 (C-21), 142.0 (C-4, C-15), 139.7 (C-6, C-11), 137.5 (C-3, C-14), 137.4 (C-20), 136.3 (C-17), 132.3 (C-8, C-13), 131.8 (C-5, C-16), 131.1 (C-7, C-12), 130.3 (C-18, C-12), 118.8 (C-19), 80.8 (C-22), 35.3 (C-9, C-10), 33.5 (C-1, C-2), 28.5 (C-23).

MS (ESI+) *m/z*: 590.3137 [(*rac*)-**B**]$^{+\bullet}$, HR-MS (ESI+) *m/z*: calculated for $C_{38}H_{42}N_2O_4$ [(*rac*)-**B**]$^{+\bullet}$: 590.3139, found: 590.3137.

Elementary analysis: calculated for $C_{38}H_{42}N_2O_4 \cdot \frac{1}{3}\,CH_2Cl_2$: C: 74.37, H: 6.95, N: 4.53, found: C: 74.55, H: 7.12, N: 4.51.

Melting point: 195 °C

R_f (cyclohexane/ethyl acetate 4:1): 0.47

UV-Vis (CH_3CN, c = 1.69 µM): λ [nm] = 253, 289.

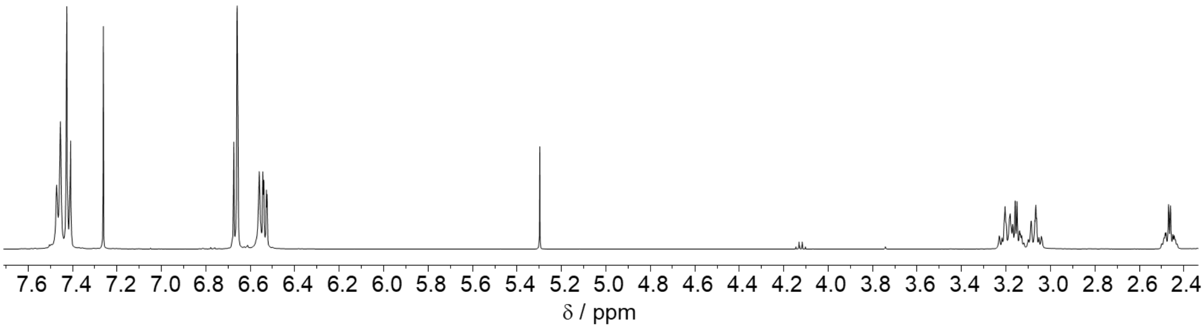

Figure S1: ^{1}H NMR spectrum (499.1 MHz, $CDCl_3$, 298 K) of (*rac*)-**B**.

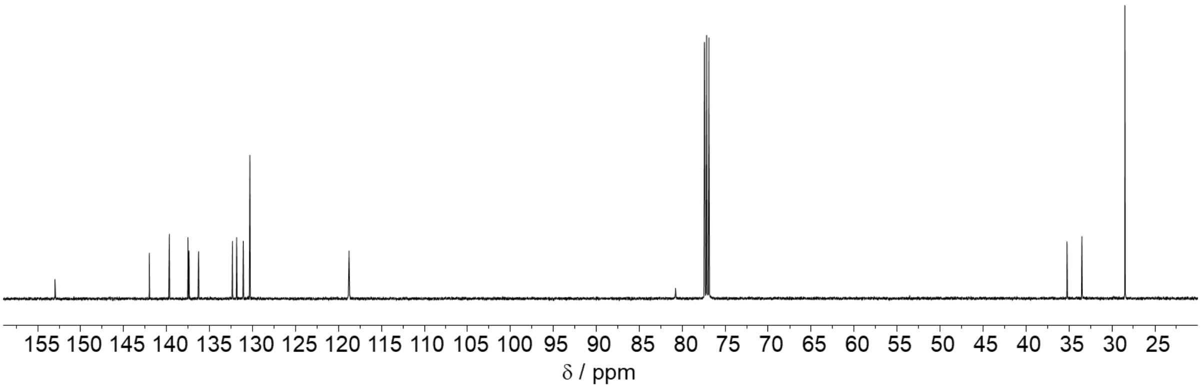

Figure S2: ^{13}C NMR spectrum (125.5 MHz, $CDCl_3$, 298 K) of (*rac*)-**B**.

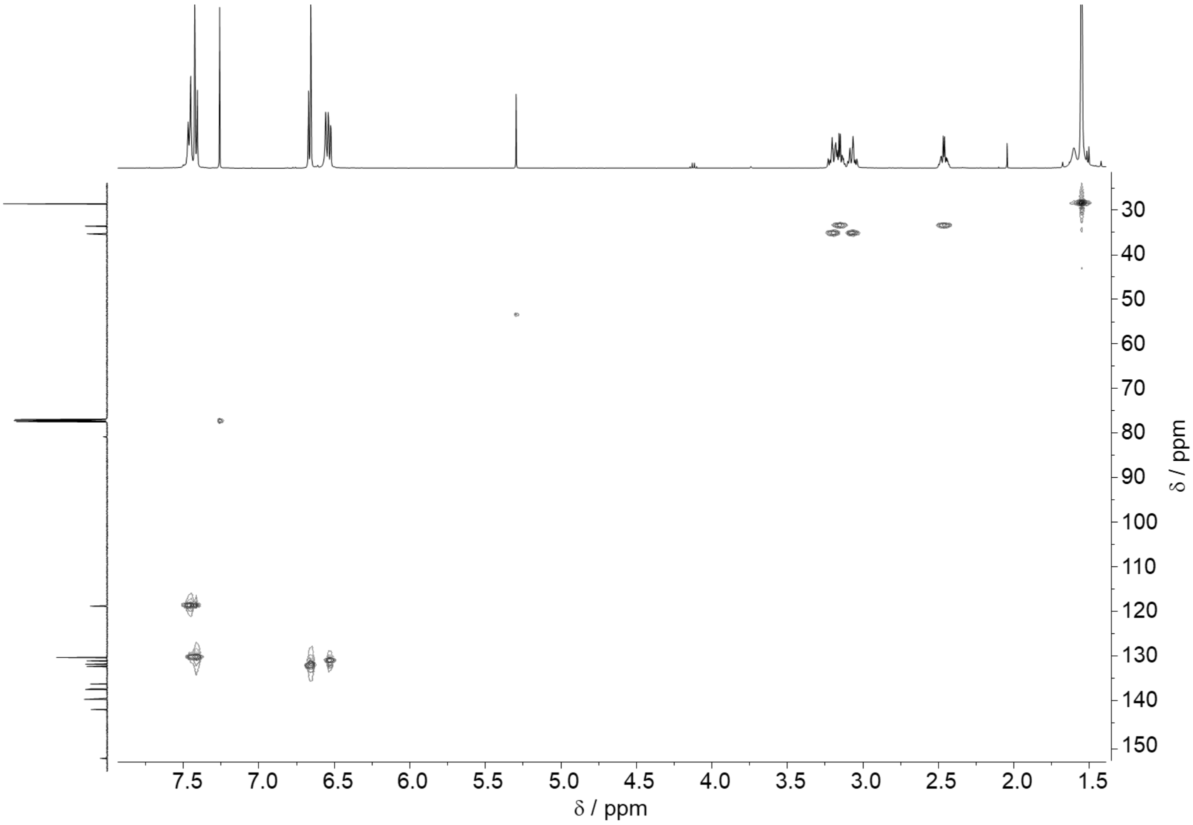

Figure S3: ^{1}H, ^{13}C-HSQC NMR spectrum (499.1 MHz, 125.5 MHz, $CDCl_3$, 298 K) of (*rac*)-**B**.

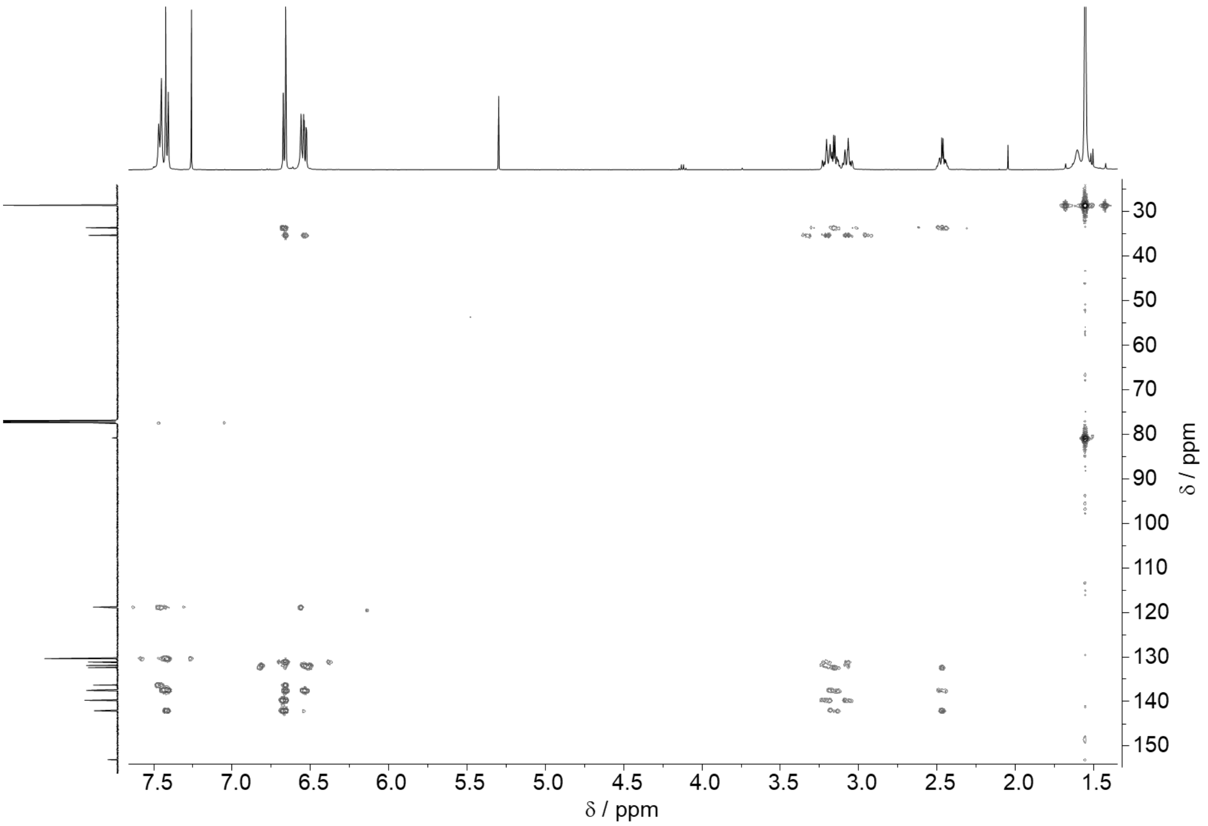

Figure S4: ^{1}H, ^{13}C-HMBC NMR spectrum (499.1 MHz, 125.5 MHz, $CDCl_3$, 298 K) of (*rac*)-**B**.

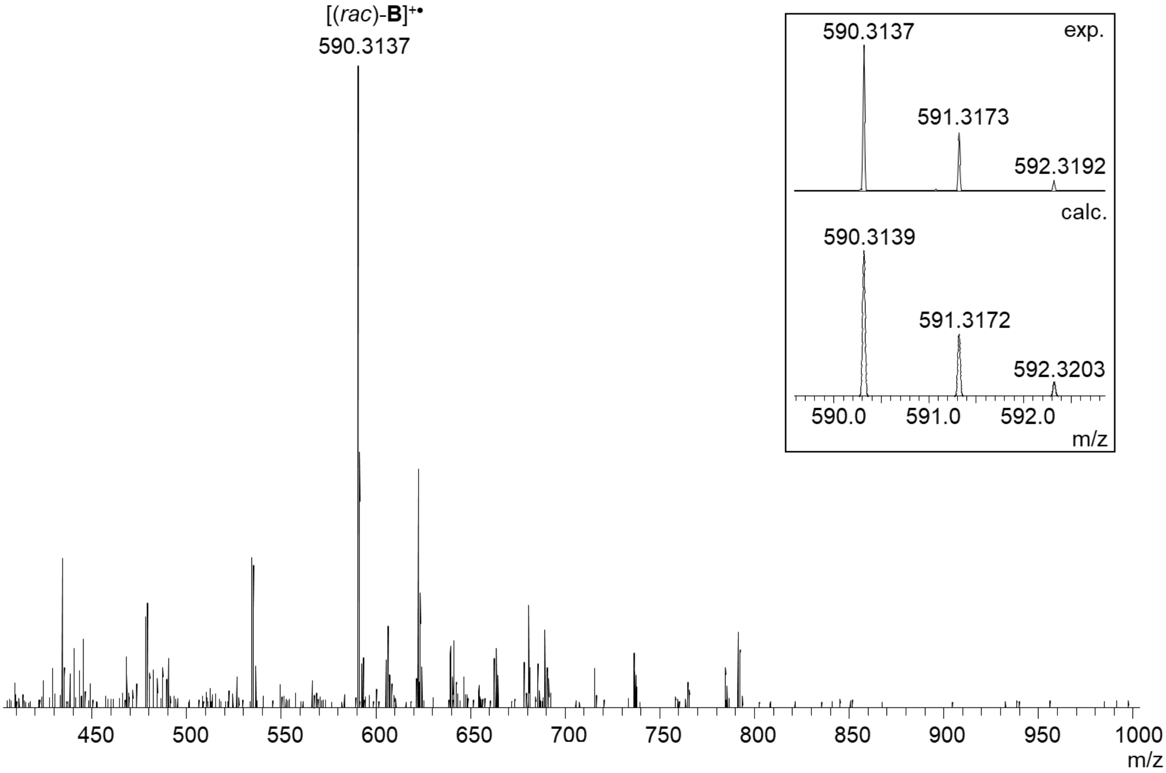

Figure S5: ESI positive mass spectrum of (*rac*)-**B**.

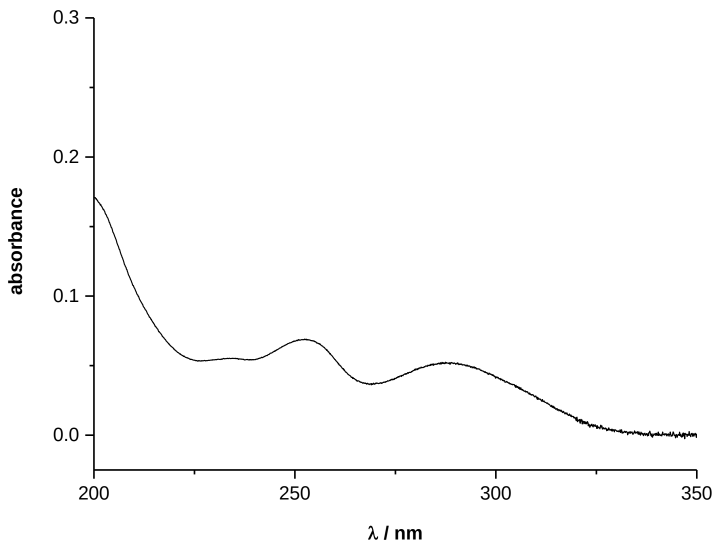

Figure S6: Non-normalized UV-Vis spectrum of (*rac*)-**B** (*c* = 1.69 µM, CH_3CN).

(*R*p)-, (*S*p)- and (*rac*)-4,15-Bis-(4-aminophenyl)[2.2]paracyclophane ((*R*p)-, (*S*p)- and (*rac*)-1)

Under an atmosphere of argon, (*rac*)-**B** (200 mg, 339 µmol, 1.00 eq.) was dissolved in dry dichloromethane (10 mL) and trifluoroacetic acid (5.22 mL, 7.73 g, 67.8 mmol, 200 eq.) was added. The reaction solution was stirred at room temperature for 18 h. Then, it was concentrated under reduced pressure, the residue was dissolved in ethyl acetate and neutralized with saturated potassium hydrogen carbonate solution. The mixture was extracted with ethyl acetate. The combined organic layers were washed with brine, dried with anhydrous magnesium sulfate and the solvent was removed under reduced pressure. The crude product was dissolved in warm acetonitrile and precipitated as hydrochloride by adding hydrochloric acid (37%, 60.0 µL, 70.8 mg, 678 µmol, 2.00 eq.). The white solid was filtered off, washed with acetonitrile and dissolved in water. After the addition of saturated potassium hydrogen solution, the mixture was extracted with ethyl acetate. The combined organic layers were washed with brine, dried with anhydrous magnesium sulfate and the solvent was removed under reduced pressure to give (*rac*)-**1** (110 mg, 282 µmol, 83%) as a yellow solid.

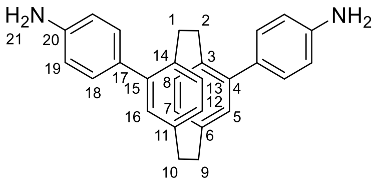

Chemical Formula: $C_{28}H_{26}N_2$
Exact Mass: 390.2096 u
Molecular Weight: 390.53 gmol^{-1}

^{1}H NMR (700.4 MHz, CD_3CN, 298 K): δ [ppm] = 7.26-7.23 (m, 4 H, H-18), 6.76-6.73 (m, 4 H, H-19), 6.63 (d, 2 H, H-8, H-13, $^3J_{8,7}$ = $^3J_{13,12}$ = 7.6), 6.61 (d, 2 H, H-5, H-16, $^4J_{5,7}$ = $^4J_{16,12}$ = 1.9 Hz), 6.54 (dd, 2 H, H-7, H-12, $^3J_{7,8}$ = $^3J_{12,13}$ = 7.6 Hz, $^4J_{7,5}$ = $^4J_{12,16}$ = 1.9 Hz), 4.23 (s, 4 H, H-21), 3.20-3.12 (m, 4H, H-1, H-2, H-9, H-10), 3.04-2.99 (m, 2 H, H-9, H-10), 2.39-2.33 (m, 2 H, H-1, H-2).

^{13}C NMR (176.1 MHz, CD_3CN, 298 K): δ [ppm] = 148.1 (C-20), 143.5 (C-4, C-15), 140.7 (C-6, C-11), 137.8 (C-3, C-14), 133.0 (C-8, C-13), 132.3 (C-5, C-16), 131.5 (C-17), 131.4 (C-18), 131.2 (C-7, C-12), 115.5 (C-19), 35.6 (C-9, C-10), 34.4 (C-1, C-2).

MS (ESI+) *m/z*: 391.2173 [**1**+H$^+$]$^+$, 196.1125 [**1**+2H$^+$]$^{2+}$; HR-MS (ESI+) *m/z*: calculated for [$C_{28}H_{26}N_2$+H]$^+$: 391.2169, found: 391.2173.

Elementary analysis: calculated for $C_{28}H_{26}N_2 \cdot \frac{1}{2}\, C_4H_8O_2$: C: 82.91, H: 6.96, N: 6.45, found: C: 83.28, H: 6.88, N: 6.40.

Melting point: 245 °C (decomposition)

R_f (cyclohexane/ethyl acetate 2:1+ 5% triethylamine): 0.19

UV-Vis (CH_3CN, c = 25.6 µM): λ [nm] = 265, 296.

HPLC analytical (*CHIRALPAK*® IB, methanol/water (95:5 v/v), flow rate 1 mL min^{-1}): (R_p)-**1** t_R = 8.38 min, (S_p)-**1** t_R = 10.82 min.

HPLC semi-preparative (*CHIRALPAK*® IB, methanol/water (95:5 v/v), 18 mLmin^{-1}): (R_p)-**1** > 99% ee, (S_p)-**1** 97% ee.

Specific rotation: (–)-(R_p)-**1**: $[\alpha]_D^{25}$ = –615° (c = 1.00 mg mL^{-1} = 2.56 mM, CH_2Cl_2), (+)-(S_p)-**1**: $[\alpha]_D^{25}$ = +646° (c = 1.00 mg mL^{-1} = 2.56 mM, CH_2Cl_2)

CD (CH_3CN, c = 256 µM): λ [nm] ($\Delta\varepsilon$ [L mol^{-1} cm^{-1}]): (R_p)-**1**: 217 (–69), 235 (+59), 248 (+13), 261 (+64), 271 (+35), 276 (+36), 313 (–110); (S_p)-**1**: 217 (+79), 235 (–65), 248 (–12), 261 (–70), 271 (–37), 276 (–38), 313 (+126).

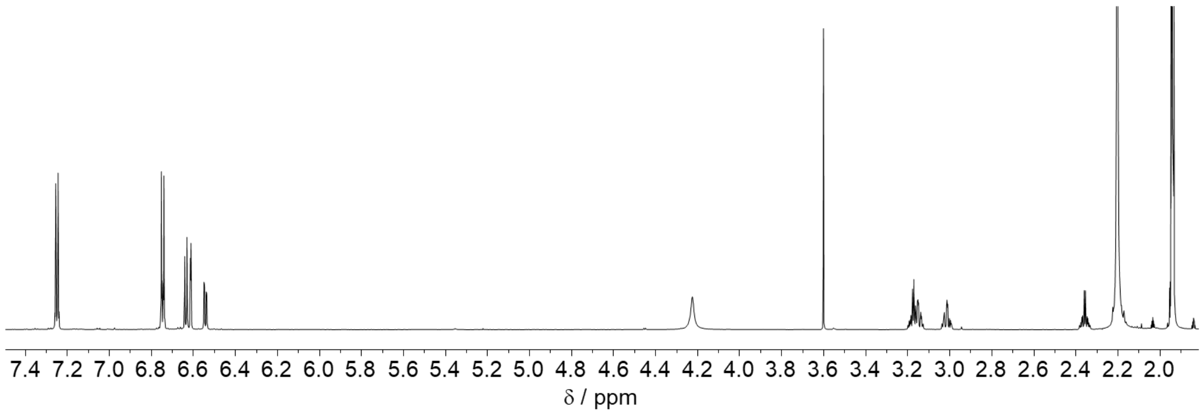

Figure S7: ^{1}H NMR spectrum (700.4 MHz, CD_3CN, 298 K) of **1**.

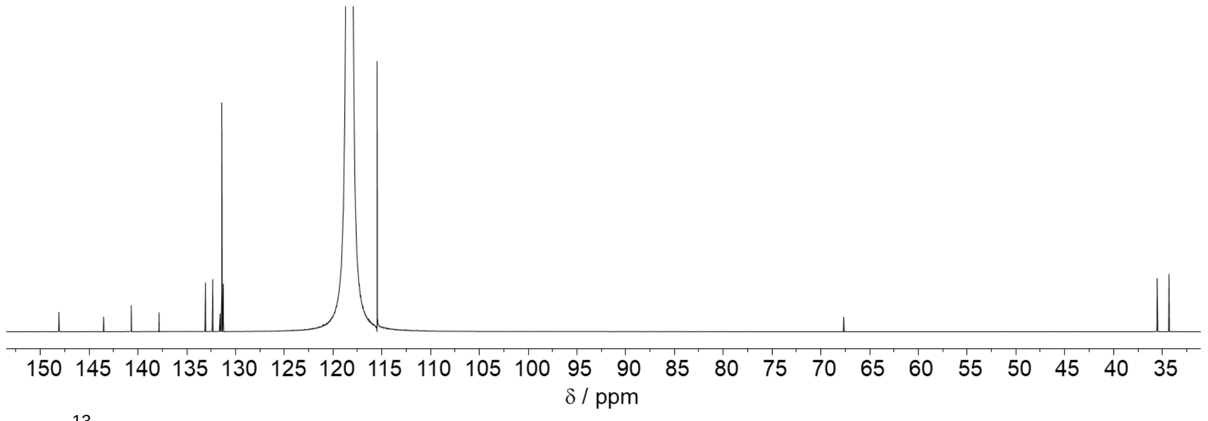

Figure S8: ^{13}C NMR spectrum (176.1 MHz, CD_3CN, 298 K) of **1**.

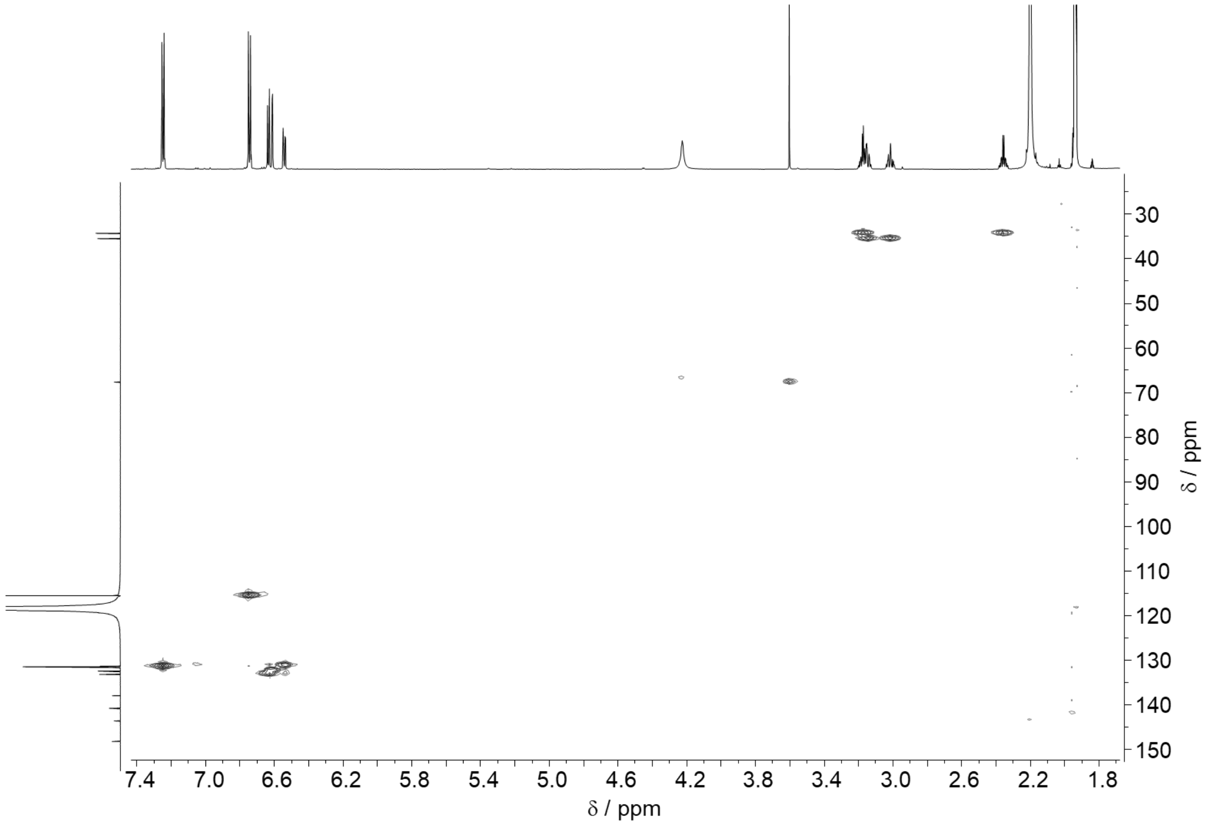

Figure S9: ^{1}H, ^{13}C-HSQC NMR spectrum (700.4 MHz, 176.1 MHz, CD_3CN, 298 K) of **1**.

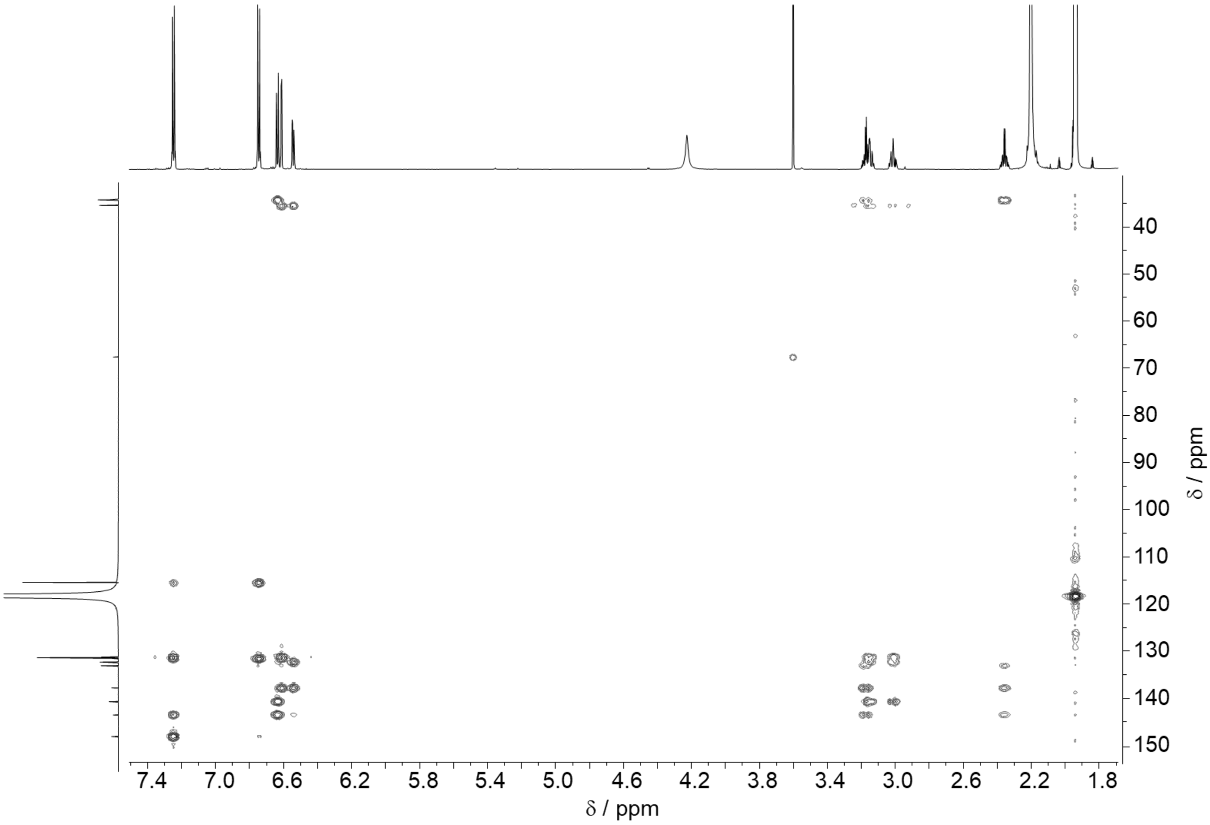

Figure S10: ^{1}H, ^{13}C-HMBC NMR spectrum (700.4 MHz, 176.1 MHz, CD_3CN, 298 K) of **1**.

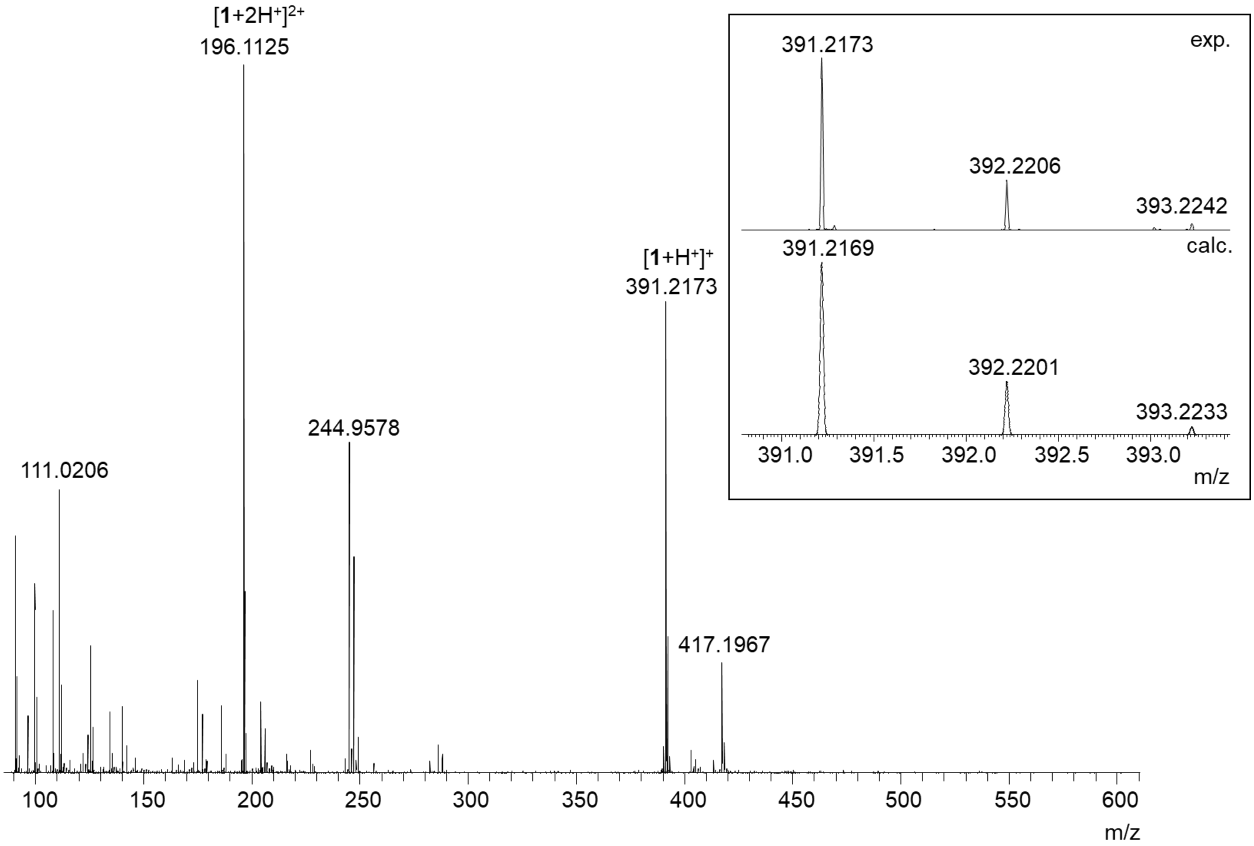

Figure S11: ESI positive mass spectrum of **1**.

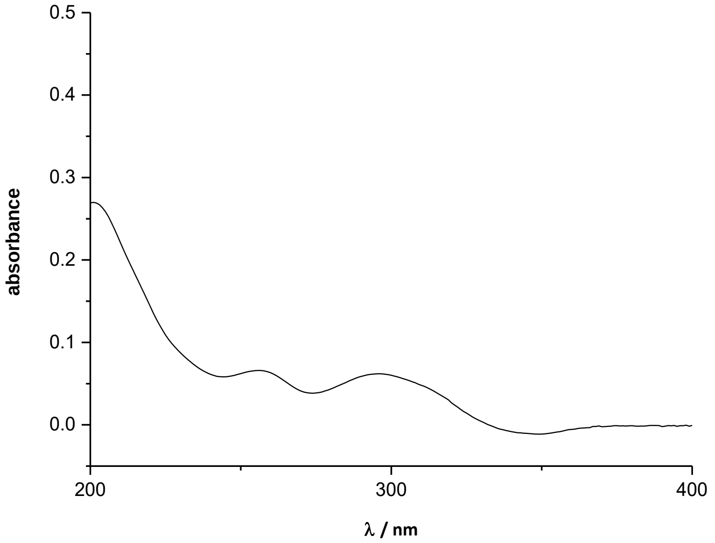

Figure S12: Non-normalized UV-Vis spectrum of **1** (c = 25.6 µM, CH_3CN).

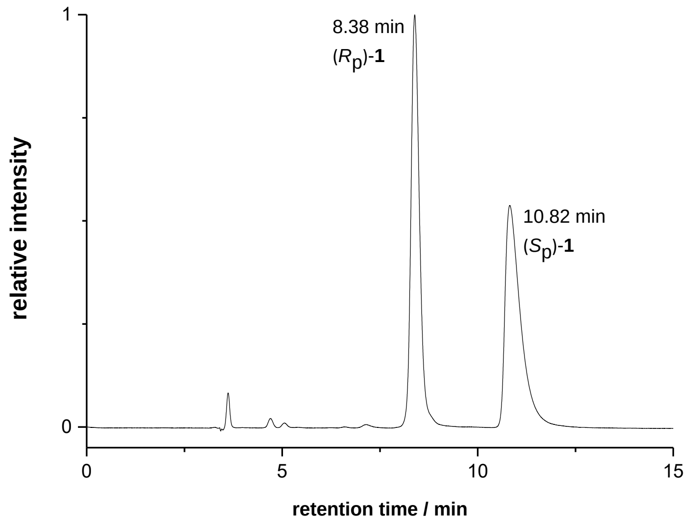

Figure S13: Chromatogram of chiral resolution of (*rac*)-**1** *via* analytical HPLC (chiral stationary phase: *CHIRALPAK*® IB column, eluent: methanol/water (95:5 v/v), flow rate: 1 mL min^{-1}). When upscaling to semi-preparative mode, the flow rate was increased to 18 mL min^{-1}.

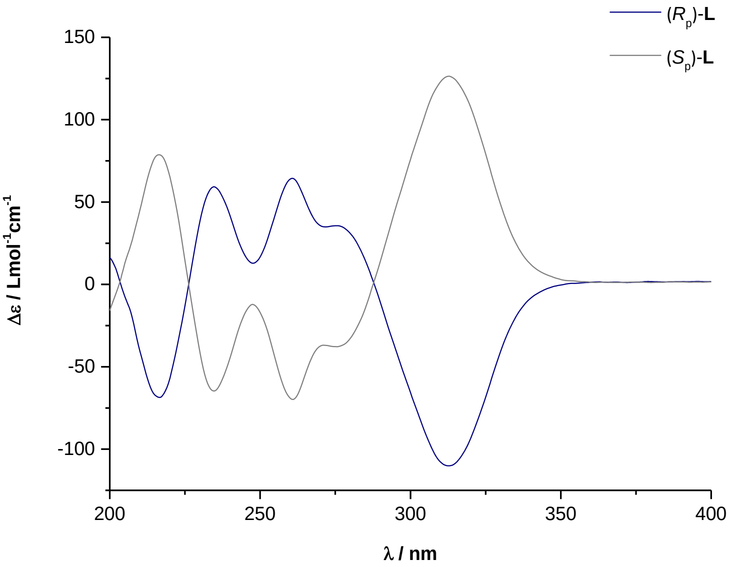

Figure S14: CD spectra of (R_p)- and (S_p)-**1** (c = 256 µM, CH_3CN).

Self-assembly and characterization of iron(II) complexes

Cyclic $[Fe_4L_6](OTf)_8$ helicates in acetonitrile

(Δ,Δ,Δ,Δ)-$[Fe_4\{(R_p)\text{-L}\}_6](OTf)_8$ or (Λ,Λ,Λ,Λ)-$[Fe_4\{(S_p)\text{-L}\}_6](OTf)_8$ obtained from (R_p)- or (S_p)-1

(R_p)- or (S_p)-**1** (3.01 mg, 7.71 µmol, 3.00 eq.) was dissolved in acetonitrile (0.6 mL) and 2-formylpyridine (1.47 µL, 1.65 mg, 15.4 µmol, 6.00 eq.) was added. The mixture was stirred at 70 °C for 30 min. After cooling to room temperature, a solution of iron(II) triflate hexahydrate (2.41 mg, 5.22 µmol, 2.03 eq.) in acetonitrile (0.1 mL) was added. The violet solution was stirred at 70 °C for 15 h. After cooling to room temperature, the solution was poured into diethyl ether (4 mL). The violet precipitate was collected and washed with diethyl ether as well as *n*-hexane and dissolved in deuterated acetonitrile (0.6 mL).

(Δ,Δ,Δ,Δ)-$[Fe_4\{(R_p)\text{-L}\}_6](OTf)_8$ and (Λ,Λ,Λ,Λ)-$[Fe_4\{(S_p)\text{-L}\}_6](OTf)_8$ obtained from (*rac*)-1

(*rac*)-**1** (4.08 mg, 10.4 µmol, 3.00 eq.) was dissolved in acetonitrile (0.6 mL) and 2-formylpyridine (1.98 µL, 2.23 mg, 20.8 µmol, 6.00 eq.) was added. The mixture was stirred at 70 °C for 30 min. After cooling to room temperature, a solution of iron(II) triflate hexahydrate (3.25 mg, 7.03 µmol, 2.03 eq.) in acetonitrile (0.6 mL) was added. The violet solution was stirred at 70 °C for 15 h. After cooling to room temperature, the solution was poured into diethyl ether (4 mL). The violet precipitate was collected and washed with diethyl ether as well as *n*-hexane and dissolved in deuterated acetonitrile (0.6 mL).

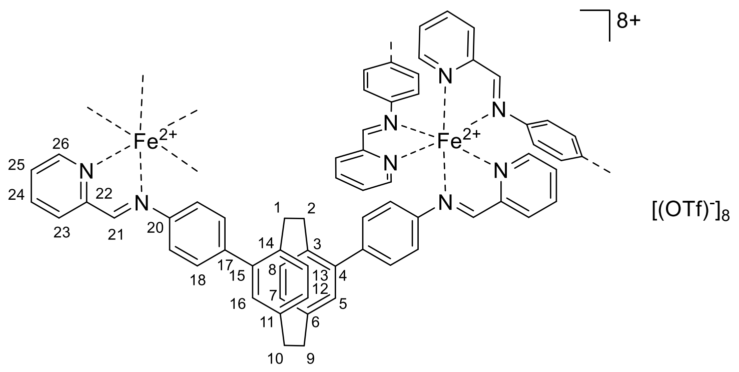

enantiomerically pure (Δ,Δ,Δ,Δ)-$[Fe_4\{(R_p)\text{-L}\}_6](OTf)_8$ and (Λ,Λ,Λ,Λ)-$[Fe_4\{(S_p)\text{-L}\}_6](OTf)_8$ obtained from (R_p)-1 or (S_p)-1, respectively

^{1}H NMR (700.4 MHz, CD_3CN, 298 K): δ[ppm] = 9.46 (s, 4 H, H-21), 9.43 (s, 4 H, H-21), 9.11 (s, 4 H, H-21), 8.78-8.73* (m, 4 H), 8.70-8.66* (m, 4 H), 8.44-8.24* (m, 20 H), 7.86-7.71* (m, 24 H), 7.67-7.62* (m, 6 H), 7.47-7.40* (m, 14 H), 7.18-7.06* (m, 12 H), 6.98-6.95* (m, 6 H), 6.83-6.79* (m, 6 H), 6.71-6.66* (m, 14 H), 6.63-6.58* (m, 8 H), 6.55-6.52* (m, 4 H), 6.45-6.37* (m, 10 H), 3.37-3.20* (m, 16 H), 3.08-2.95* (m, 12 H), 2.87-2.82* (m, 4 H), 2.80-2.74* (m, 4 H), 2.50-2.45* (m, 4 H), 2.08-2.05* (m, 4 H), 1.90-1.84* (m, 4 H).

^{13}C NMR (176.1 MHz, CD_3CN, 298 K): δ [ppm] = 174.7 (C-21), 173.9 (C-21), 173.4 (C-21), 163.4*, 160.0*, 159.8*, 159.0*, 158.5*, 157.9*, 157.2*, 152.9*, 152.2*, 147.3*, 146.2*, 143.2*, 143.1*, 142.2*, 141.8*, 141.8*, 141.7*, 141.4*, 141.3*, 141.2*, 140.1*, 139.9*, 139.1*, 138.4*, 137.5*, 137.3*, 133.5*, 133.4*, 133.2*, 133.2*, 133.1*, 132.8*, 132.7*, 132.3*, 132.2*, 131.8*, 131.3*, 131.1*, 130.0*, 129.7*, 129.7*, 126.0*, 124.1*, 124.1*, 123.2*, 35.5*, 35.4*, 35.2*, 34.3*, 34.2*, 33.5*.

*signals could not be unambiguously assigned.

^{1}H-2D-DOSY NMR (700.4 MHz, CD_3CN, 298 K): $D = 4.94 \cdot 10^{-10}$ m^2 s^{-1}, R_H = 13.7 Å.

MS (ESI+) *m/z*: 374.1656 [**1** + 4 formylpyridine – 4 H_2O + $2H^+$]$^{2+}$: $C_{52}H_{40}N_6^{2+}$, 540.6048 {[Fe_4{(R_p)-**L**}$_6$]OTf}$^{7+}$, 655.5365 {[Fe_4{(R_p)-**L**}$_6$](OTf)$_2$}$^{6+}$, 773.1460 {[Fe_2{(R_p)-**L**}$_2$](OTf)$_2$}$^{2+}$, 816.6298 {[Fe_4{(R_p)-**L**}$_6$](OTf)$_3$}$^{5+}$, 844.9554 {[Fe_2{(R_p)-**L**}$_4$](OTf)}$^{3+}$, 1057.7751 {[Fe_4{(R_p)-**L**}$_6$](OTf)$_4$}$^{4+}$, 1460.0054 {[Fe_4{(R_p)-**L**}$_6$](OTf)$_5$}$^{3+}$, 2264.9084 {[Fe_4{(R_p)-**L**}$_6$](OTf)$_6$}$^{2+}$.

UV-Vis (CH_3CN, c = 2.14 mM): λ [nm] = 224, 290, 362, 580.

CD (CH_3CN, c = 2.14 mM): λ [nm] ($\Delta\varepsilon$ [L mol^{-1} cm^{-1}]): (Δ,Δ,Δ,Δ)-[Fe_4{(R_p)-**L**}$_6$](OTf)$_8$: 214 (–116), 247 (+183), 288 (–180), 323 (–117), 357 (–149), 438 (–12), 510 (–96), 608 (+160); (Λ,Λ,Λ,Λ)-[Fe_4{(S_p)-**L**}$_6$](OTf)$_8$: 214 (+77), 247 (–126), 288 (+128), 325 (+90), 348 (+110), 438 (+29), 515 (+78), 606 (–85).

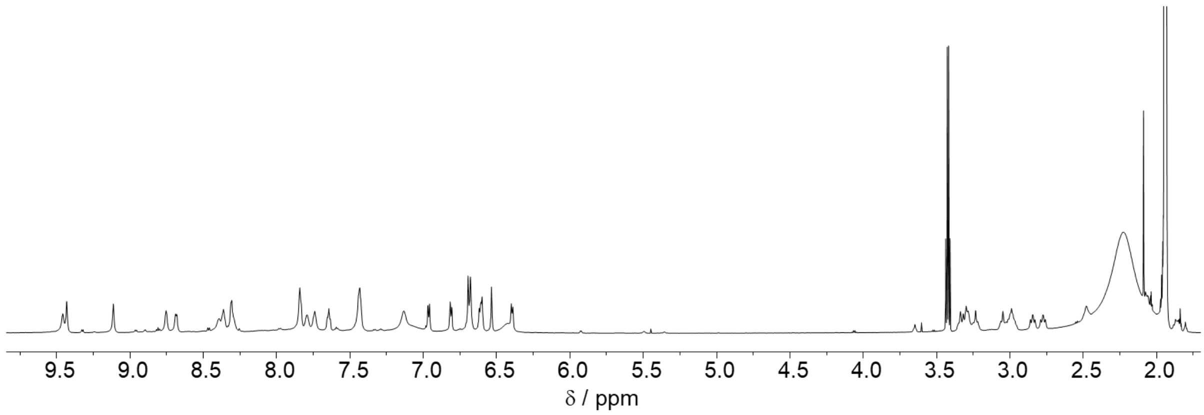

Figure S15: ^{1}H NMR spectrum (700.4 MHz, CD_3CN, 298 K) of (Δ,Δ,Δ,Δ)-[Fe_4{(R_p)-**L**}$_6$](OTf)$_8$.

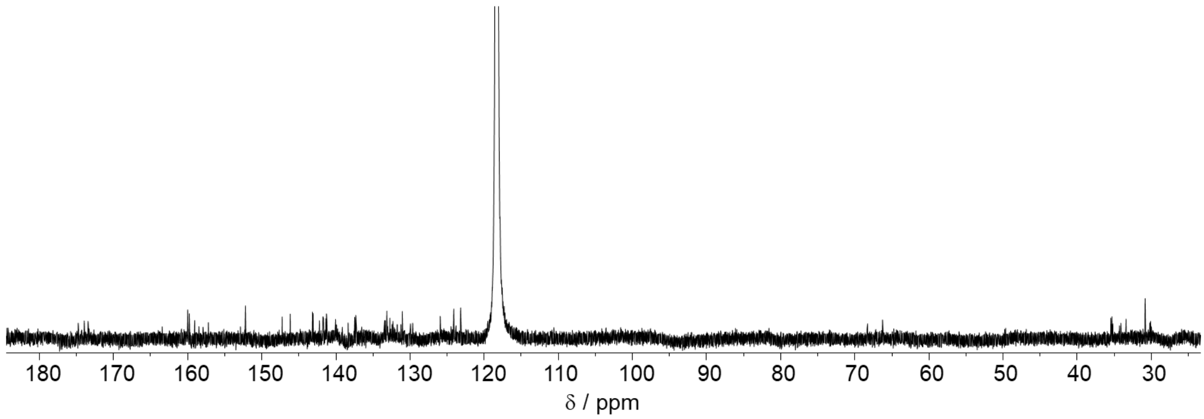

Figure S16: ^{13}C NMR spectrum (176.1 MHz, CD_3CN, 298 K) of (Δ,Δ,Δ,Δ)-[Fe_4{(R_p)-**L**}$_6$](OTf)$_8$.

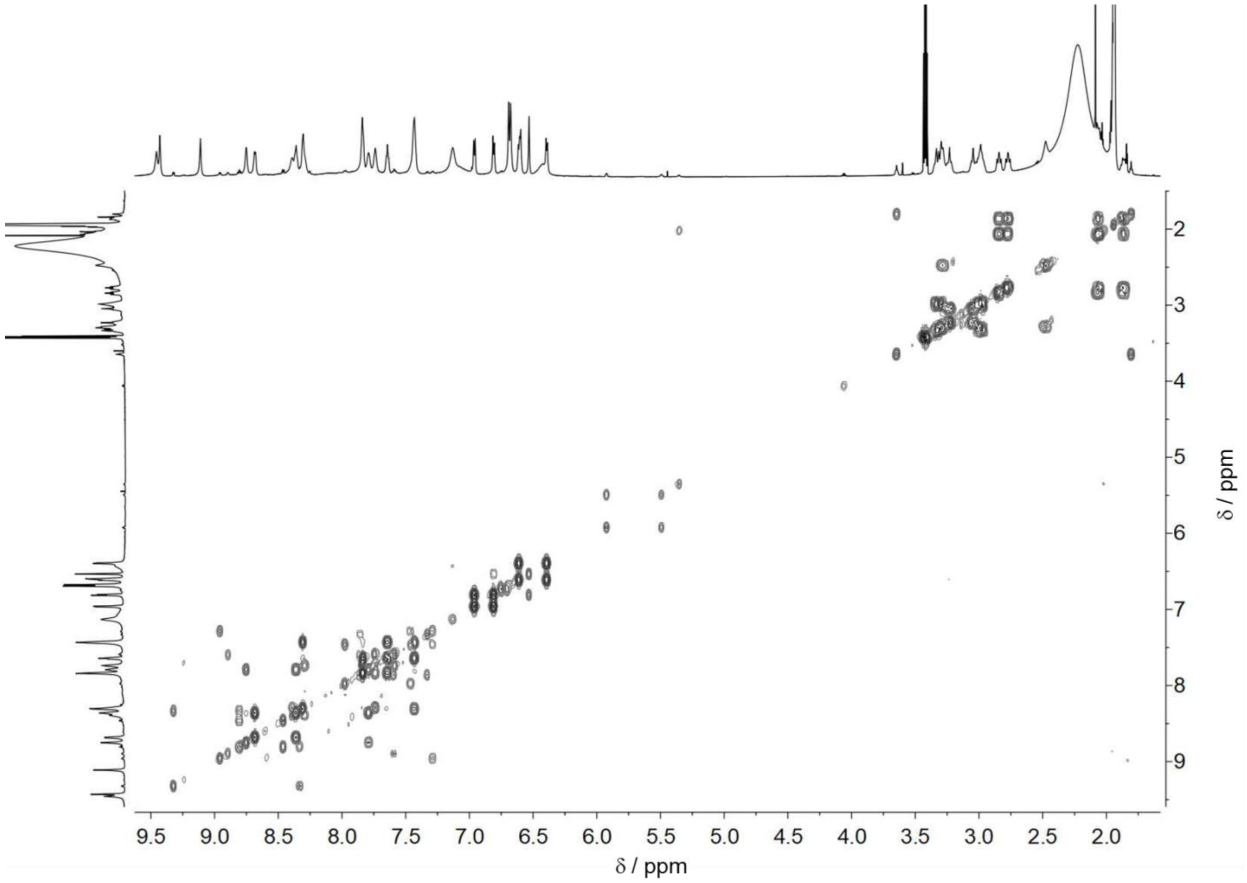

Figure S17: ^{1}H, ^{1}H-COSY NMR spectrum (700.4 MHz, CD_3CN, 298 K) of (Δ,Δ,Δ,Δ)-[Fe_4{(R_p)-**L**}$_6$]($OTf)_8$.

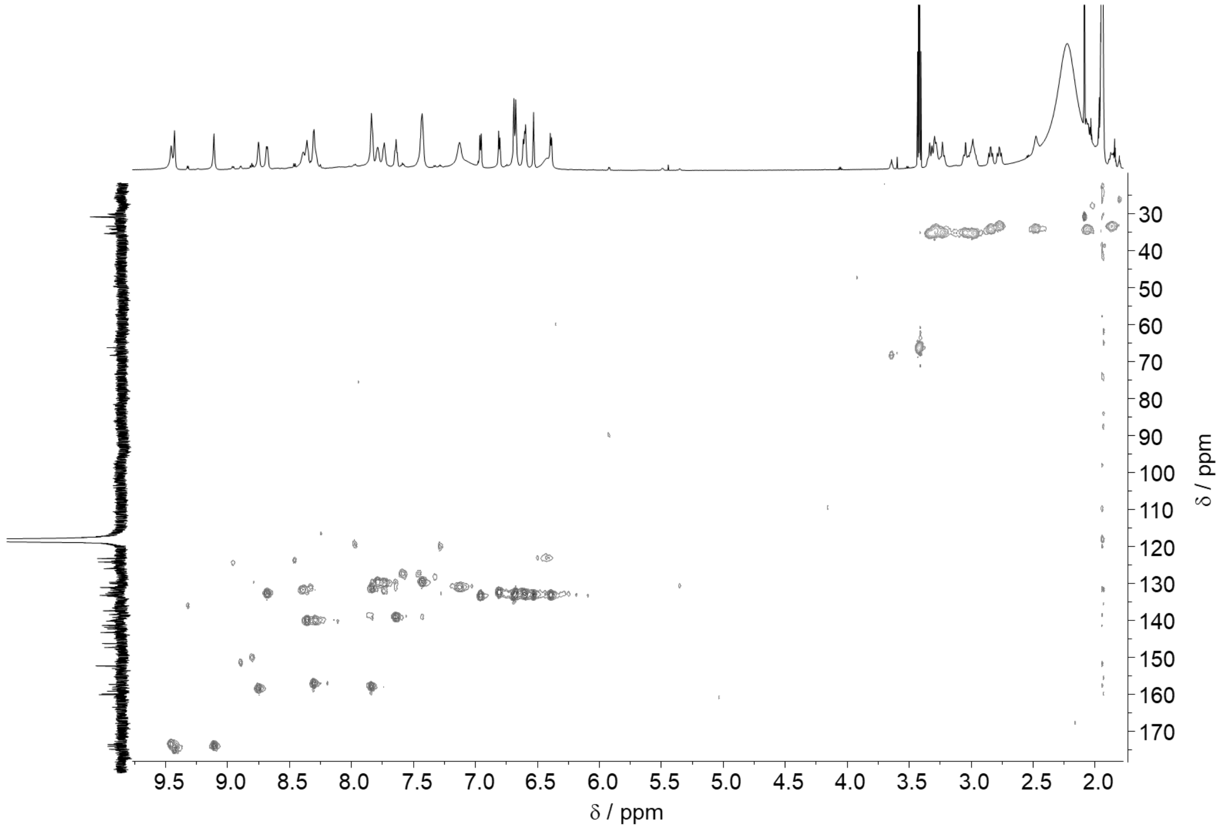

Figure S18: ^{1}H, ^{13}C-HSQC NMR spectrum (700.4 MHz, 176.1 MHz, CD_3CN, 298 K) of (Δ,Δ,Δ,Δ)-[Fe_4{(R_p)-**L**}$_6$]($OTf)_8$.

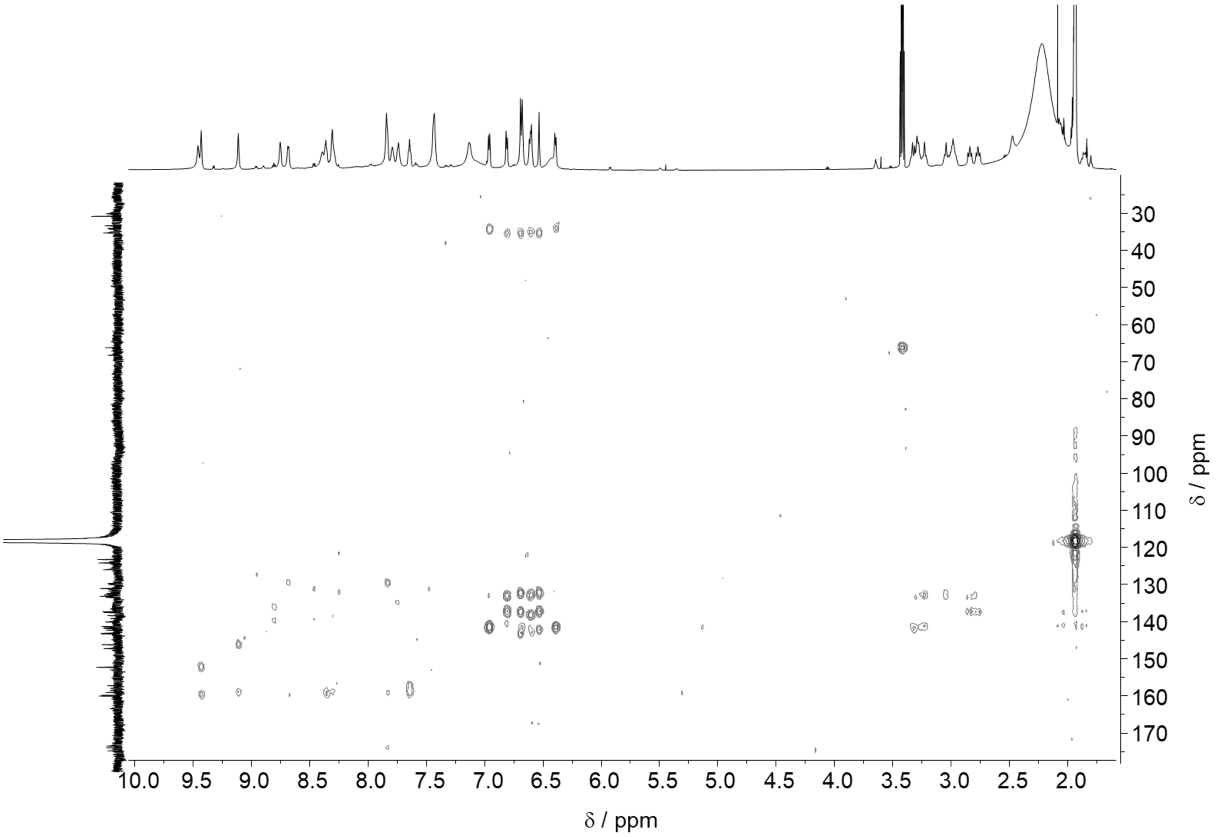

Figure S19: ^{1}H, ^{13}C-HMBC NMR spectrum (700.4 MHz, 176.1 MHz, CD_3CN, 298 K) of (Δ,Δ,Δ,Δ)-[Fe_4{(R_p)-**L**}$_6$]$(OTf)_8$.

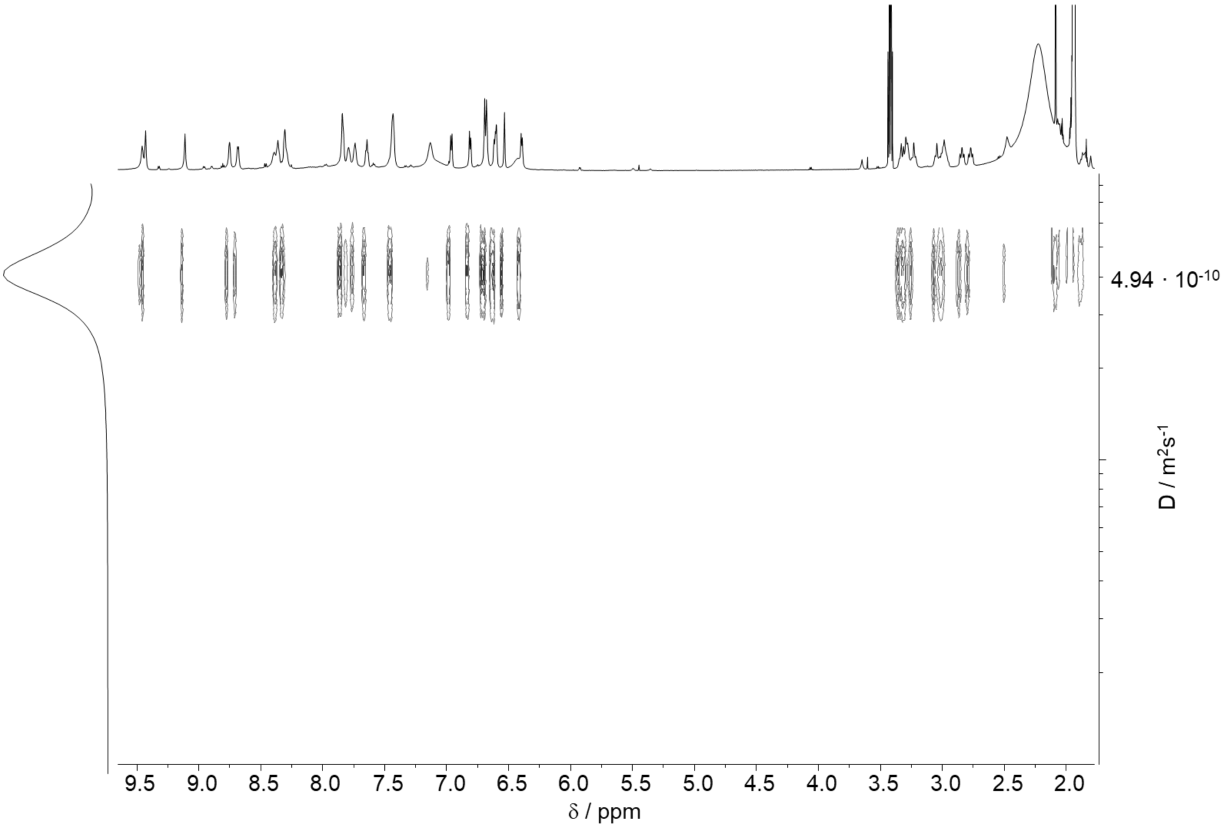

Figure S20: ^{1}H-2D-DOSY NMR spectrum (700.4 MHz, CD_3CN, 298 K) of (Δ,Δ,Δ,Δ)-[Fe_4{(R_p)-**L**}$_6$]$(OTf)_8$.

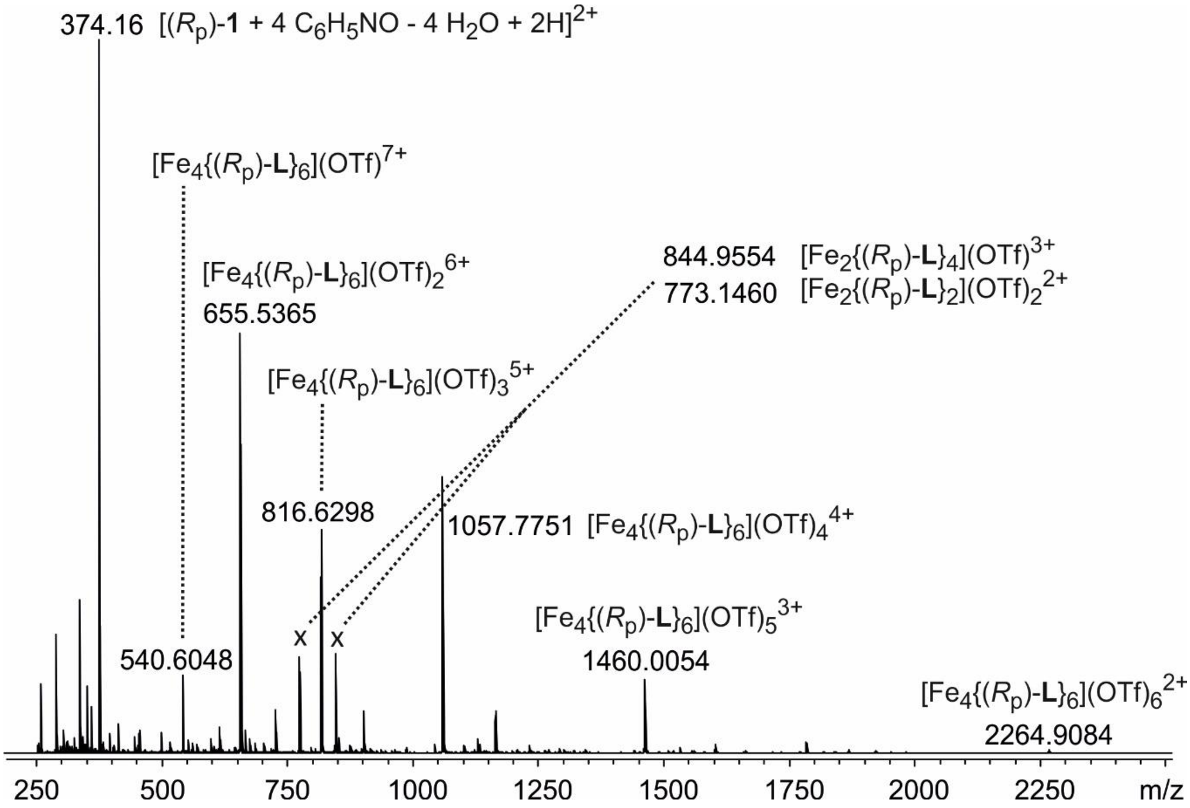

Figure S21: ESI positive mass spectrum of (Δ,Δ,Δ,Δ)-[Fe_4{(R_p)-**L**}$_6$](OTf)$_8$ in CD_3CN measured on a *micrOTOF-Q* quadrupole/time-of-flight spectrometer with ESI parameters tuned to very soft ionization conditions. An accurate mass determination of *m/z* 374.16 at the Orbitrap XL spectrometer resulted in *m/z* 374.1656. A putative structure is given below. The ESI response of this stable dication is supposed to be extremely high and thus a strong signal is expected even if the ion is present in very low concentration. According to CID-MS^2 experiments, the signals at *m/z* 773 and *m/z* 845 marked with x are gas-phase fragments of the very labile {[Fe_4{(R_p)-**L**}$_6$](OTf)$_3$}$^{5+}$ at *m/z* 816.

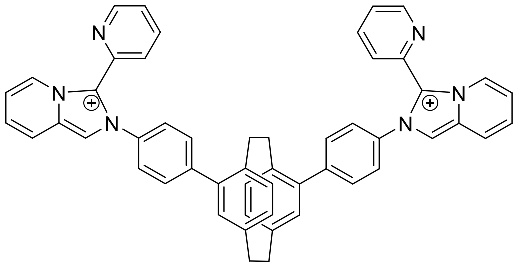

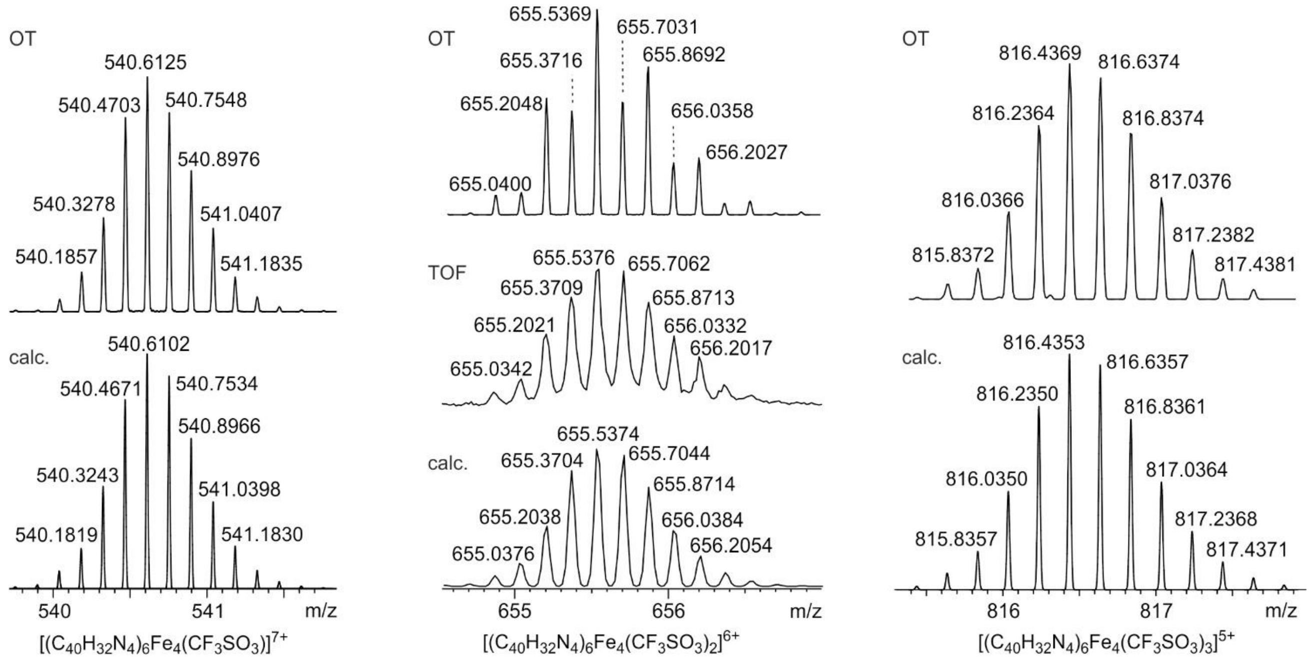

Figure S22: Experimental and calculated high resolution ESI positive mass spectra of (Δ,Δ,Δ,Δ)-[Fe_4{(R_p)-**L**}$_6$]($OTf)_8$ in CD_3CN measured on an *Orbitrap XL* mass spectrometer (OT) or a *micrOTOF-Q* time-of-flight spectrometer (TOF).

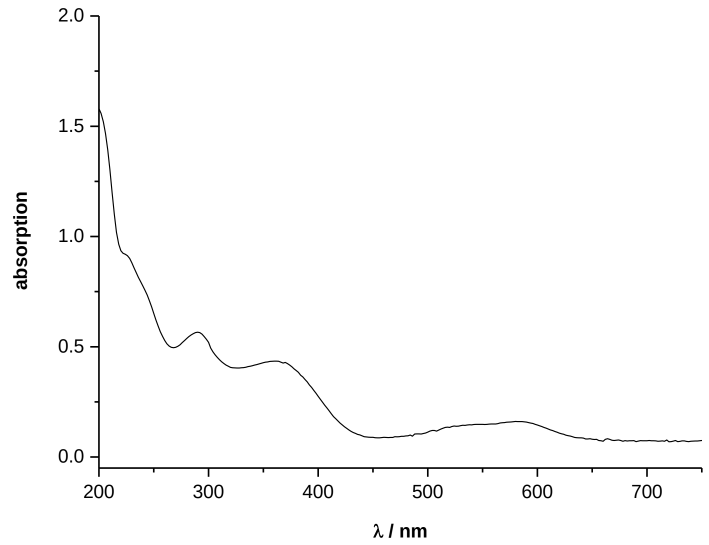

Figure S23: Non-normalized UV-Vis spectrum of [Fe_4**L**$_6$]($OTf)_8$ (c = 2.14 mM in CH_3CN).

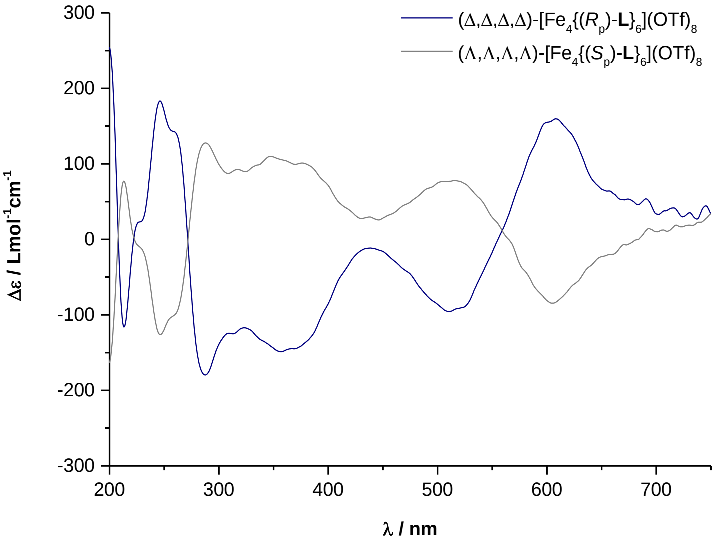

Figure S24: CD spectra of (Δ,Δ,Δ,Δ)-$[Fe_4\{(R_p)\text{-}\mathbf{L}\}_6](OTf)_8$ and (Λ,Λ,Λ,Λ)-$[Fe_4\{(S_p)\text{-}\mathbf{L}\}_6](OTf)_8$ (c = 2.14 mM in CH_3CN).

racemic mixture of (Δ,Δ,Δ,Δ)-$[Fe_4\{(R_p)\text{-}L\}_6](OTf)_8$ and (Λ,Λ,Λ,Λ)-$[Fe_4\{(S_p)\text{-}L\}_6](OTf)_8$ obtained as the major product from (*rac*)-1

^{1}H NMR (700.1 MHz, CD_3CN, 298 K): δ[ppm] = 9.46 (s, 4 H, H-21), 9.43 (s, 4 H, H-21), 9.11 (s, 4 H, H-21), 8.77-8.74* (m, 4 H), 8.70-8.67* (m, 4 H), 8.44-8.24* (m, 20 H), 7.86-7.71* (m, 24 H), 7.67-7.62* (m, 6 H), 7.46-7.41* (m, 14 H), 7.17-7.11* (m, 12 H), 6.98-6.95* (m, 6 H), 6.83-6.79* (m, 6 H), 6.71-6.66* (m, 14 H), 6.63-6.58* (m, 8 H), 6.55-6.52* (m, 4 H), 6.41-6.38* (m, 10 H), 3.40-3.20* (m, 16 H), 3.08-2.95* (m, 12 H), 2.87-2.81* (m, 4 H), 2.80-2.74* (m, 4 H), 2.50-1.84* (m, 12 H).

*Signals could not be unambiguously assigned.

^{1}H 2D-DOSY NMR (499.1 MHz, CD_3CN, 298 K): $D = 3.90\cdot10^{-10}$ m^2 s^{-1}, R_H = 14.4 Å.

MS (ESI+) *m/z*: 374.162 [**1** + 4 formylpyridine – 4 H_2O + $2H^+$]$^{2+}$: $C_{52}H_{40}N_6^{2+}$, 454.410 $[Fe_2\mathbf{L}_3]^{4+}$, 504.421 $\{Fe_2\mathbf{X}\}^{4+}$, 655.534 $\{[Fe_2\mathbf{L}_3]OTf\}^{3+}$ and $\{[Fe_4\mathbf{L}_6](OTf)_2\}^{6+}$, 658.297 [**1** + 3 formylpyridine – 3 H_2O + H^+]$^+$: $C_{46}H_{36}N_5^+$, 722.214 $\{[Fe_2\mathbf{X}](OTf)\}^{3+}$, 773.143 $\{[Fe_2\mathbf{L}_2](OTf)_2\}^{2+}$, 816.429 $\{[Fe_4\mathbf{L}_6](OTf)_3\}^{5+}$, 1057.768 $\{[Fe_4\mathbf{L}_6](OTf)_4\}^{4+}$ and $\{[Fe_2\mathbf{L}_3](OTf)_2\}^{2+}$, 1157.786 $\{[Fe_2\mathbf{X}](OTf)_2\}^{2+}$, 1460.336 $\{[Fe_4\mathbf{L}_6](OTf)_5\}^{3+}$.

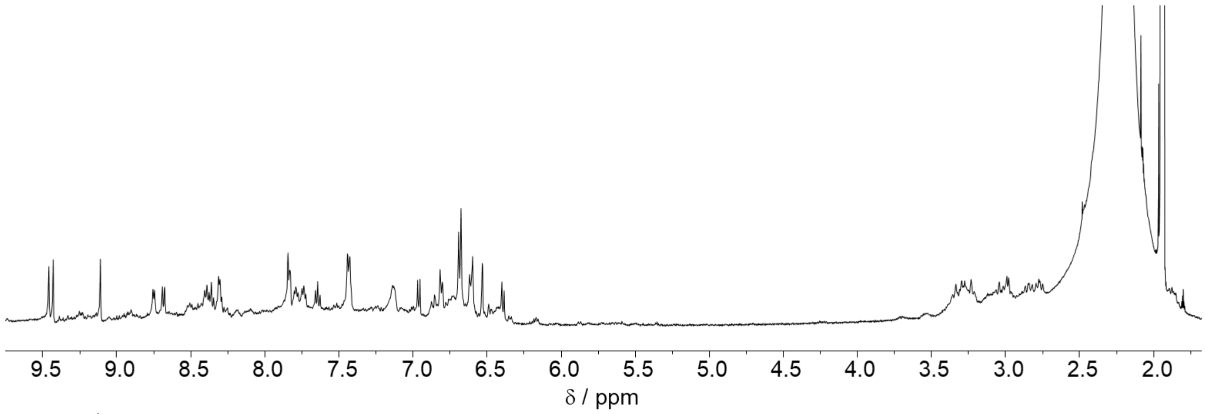

Figure S25: ^{1}H NMR spectrum (700.4 MHz, CD_3CN, 298 K) of the product of the subcomponent self-assembly process of (*rac*)-**1**, 2-formylpyridine and iron(II) triflate hexahydrate.

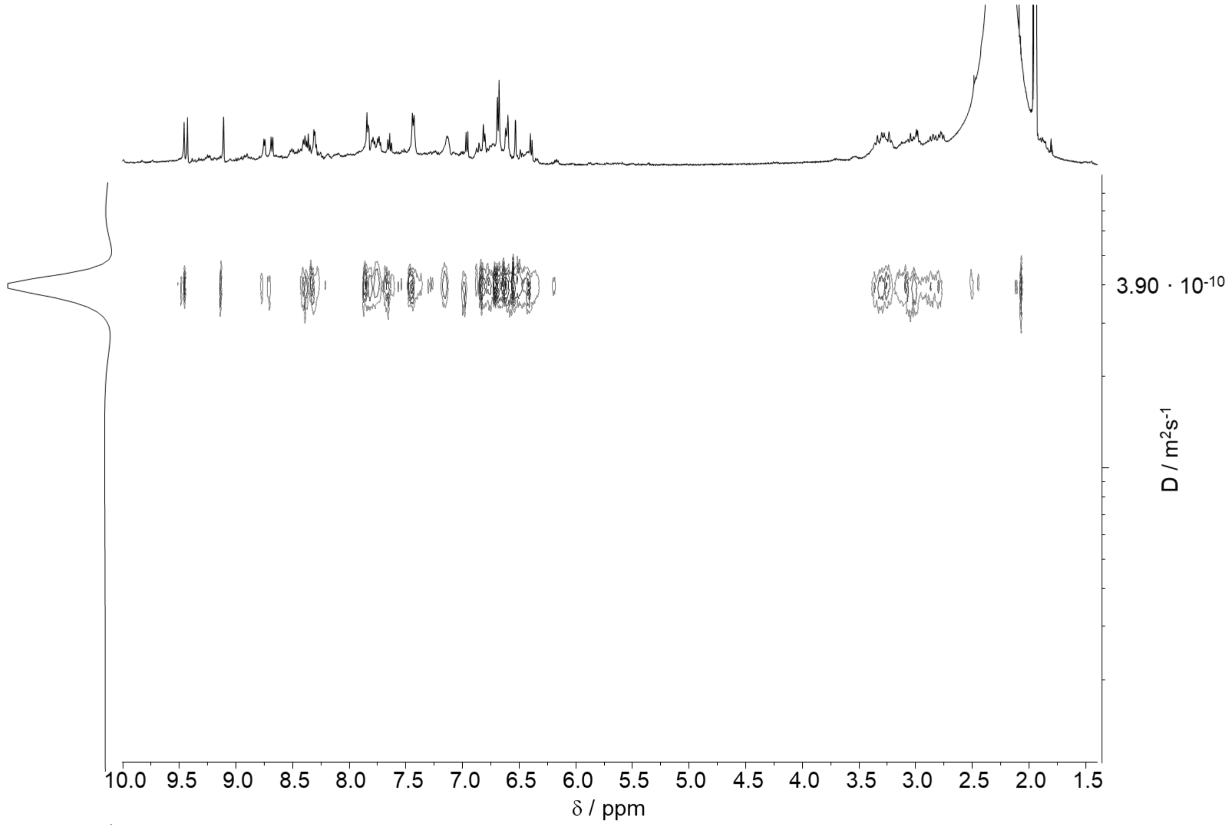

Figure S26: ^{1}H 2D-DOSY NMR spectrum (700.4 MHz, CD_3CN, 298 K) of the product of the subcomponent self-assembly process of (*rac*)-**1**, 2-formylpyridine and iron(II) triflate hexahydrate.

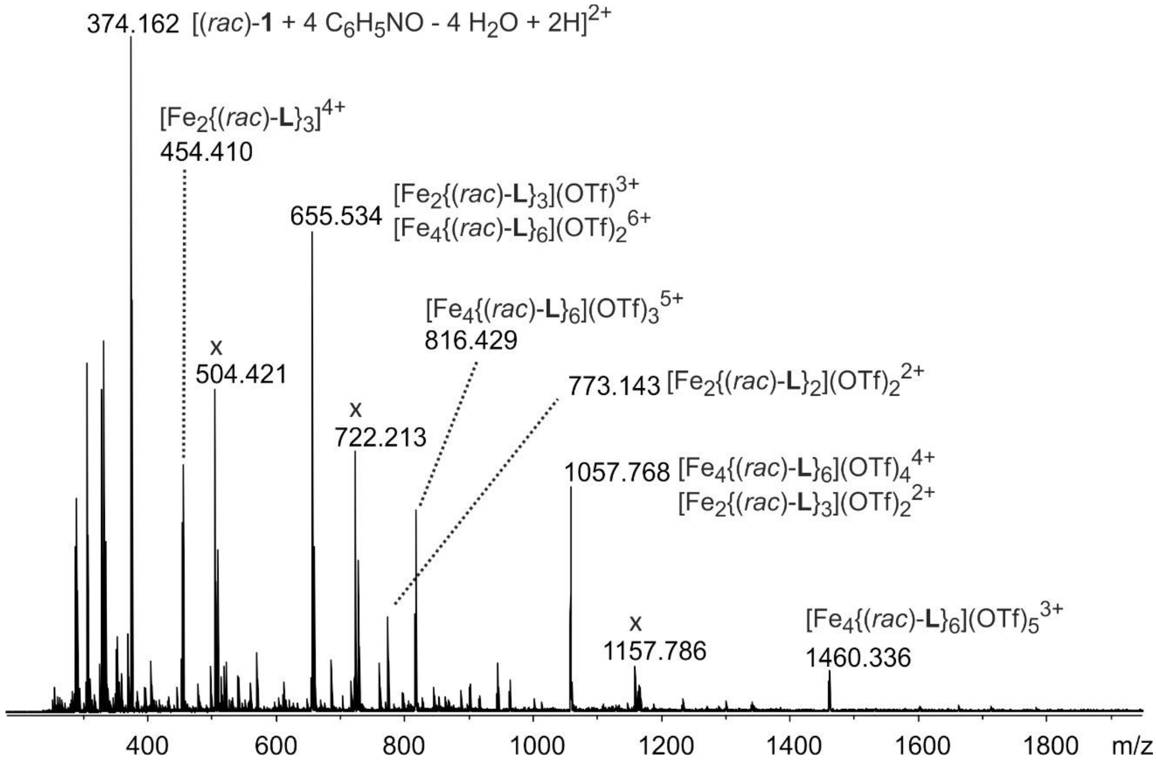

Figure S27: ESI positive mass spectrum of a 3:6:2 mixture of (*rac*)-**1**, 2-formylpyridine and $Fe(OTf)_2$ in CD_3CN measured on a *micrOTOF-Q* quadrupole/time-of-flight spectrometer with ESI parameters tuned to very soft ionization conditions. A putative structure for the signal at *m/z* 374.2^{2+} is given in Figure S21. The signals marked with X (*m/z* 504.4^{4+}, 722.2^{3+}, 1157.8^{2+}) belong to a dinuclear iron complex with triflate anions.

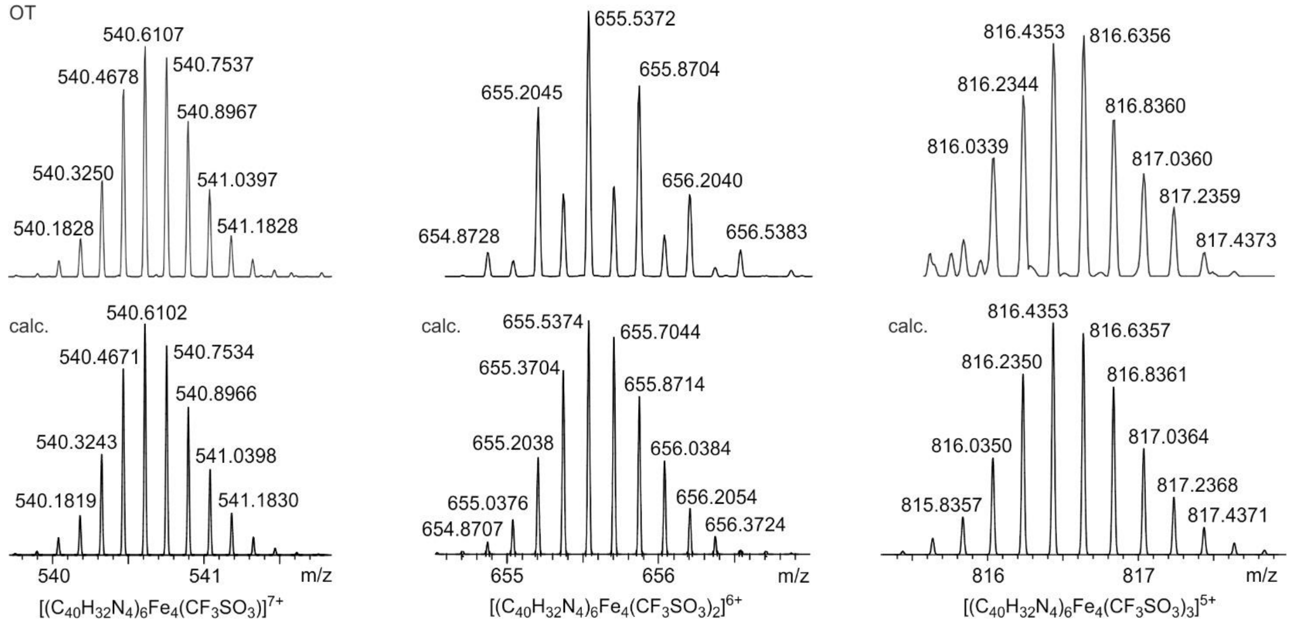

Figure S28: Experimental and calculated high resolution ESI positive mass spectra of a 3:6:2 mixture of (*rac*)-**1**, 2-formylpyridine and $Fe(OTf)_2$ in CD_3CN measured on a *Orbitrap XL* spectrometer.

Schematic lists of possible diastereomers of cyclic tetranuclear aggregates

Table S1: Schematic drawings of possible aggregates formed from enantiomerically pure starting material

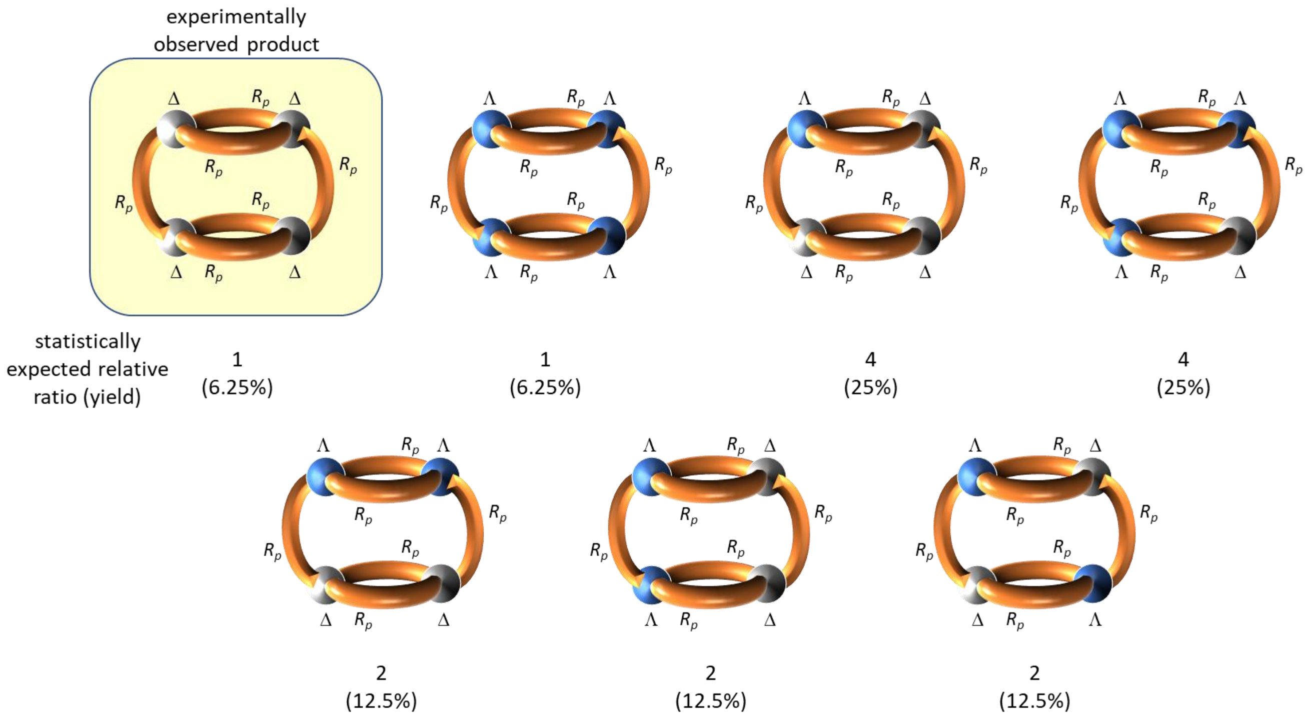

Table S2: Schematic drawings of possible aggregates formed from racemic starting material

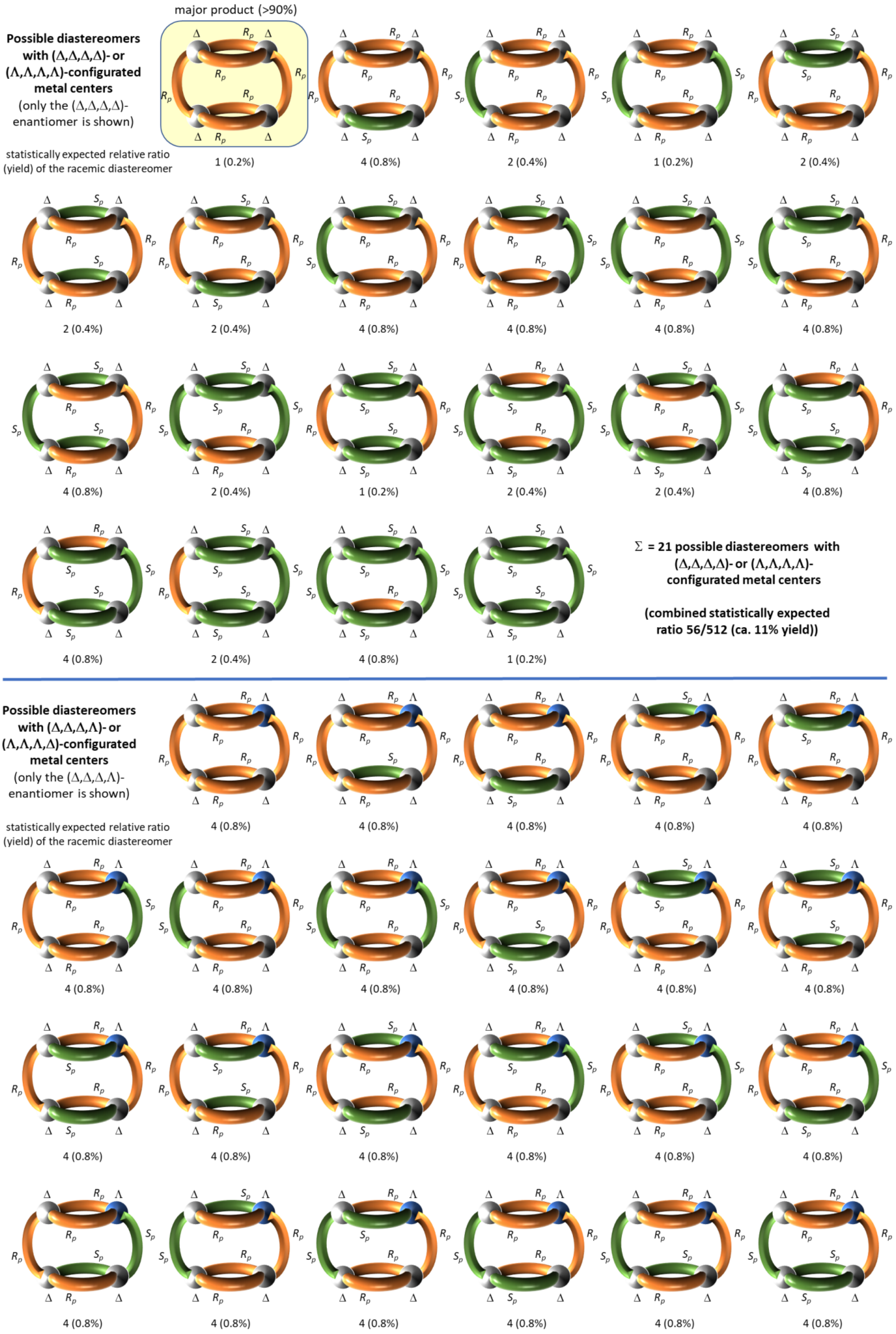

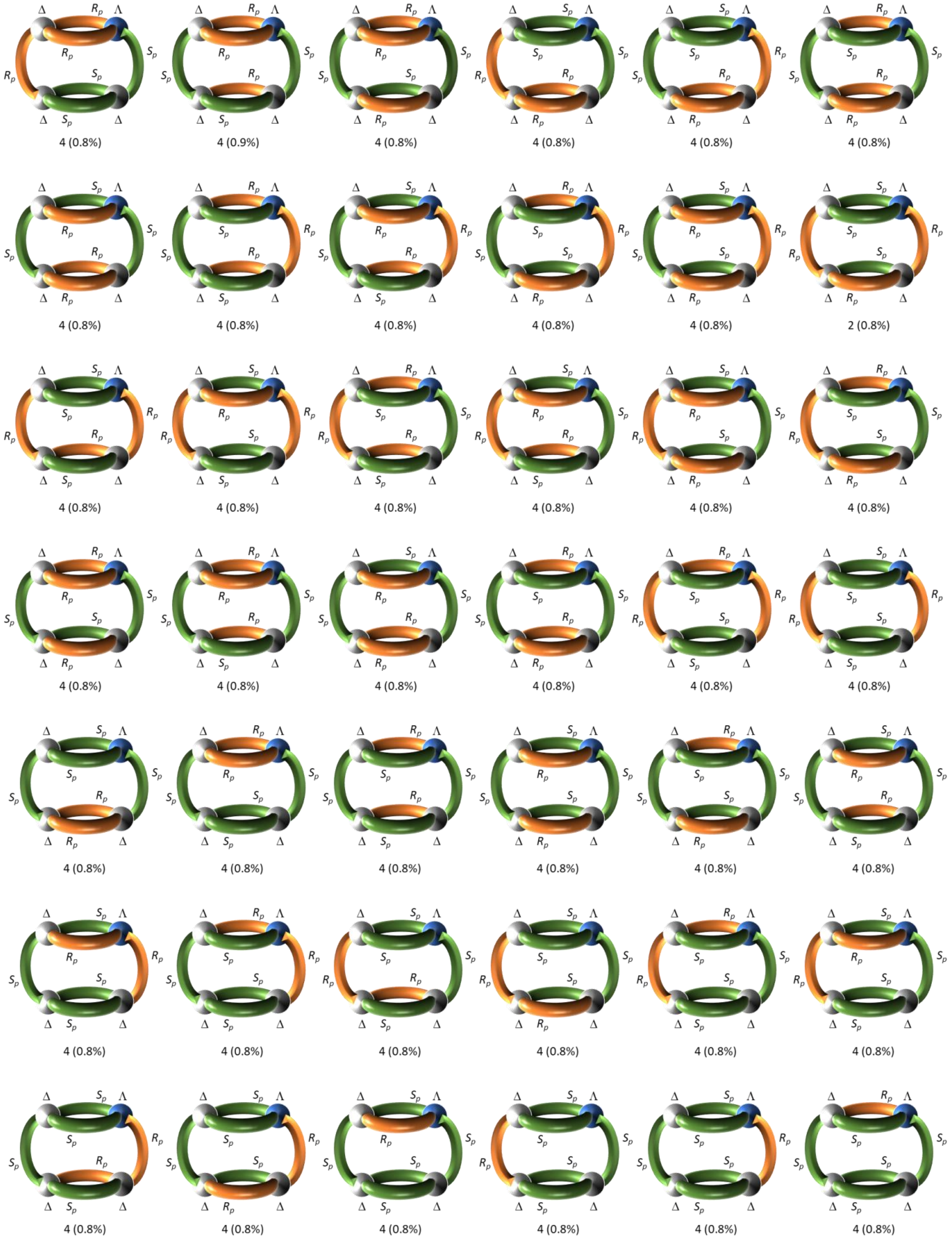

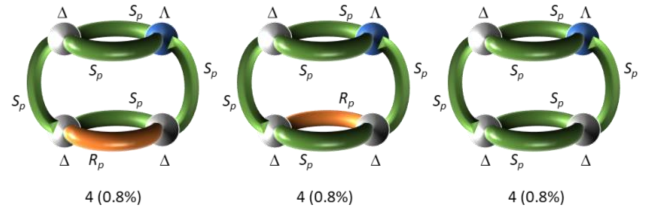

Σ = 68 possible diastereomers with (Δ,Δ,Δ,Λ)- or (Λ,Λ,Λ,Δ)-configurated metal centers

(combined statistically expected ratio 272/512 (ca. 53% yield))

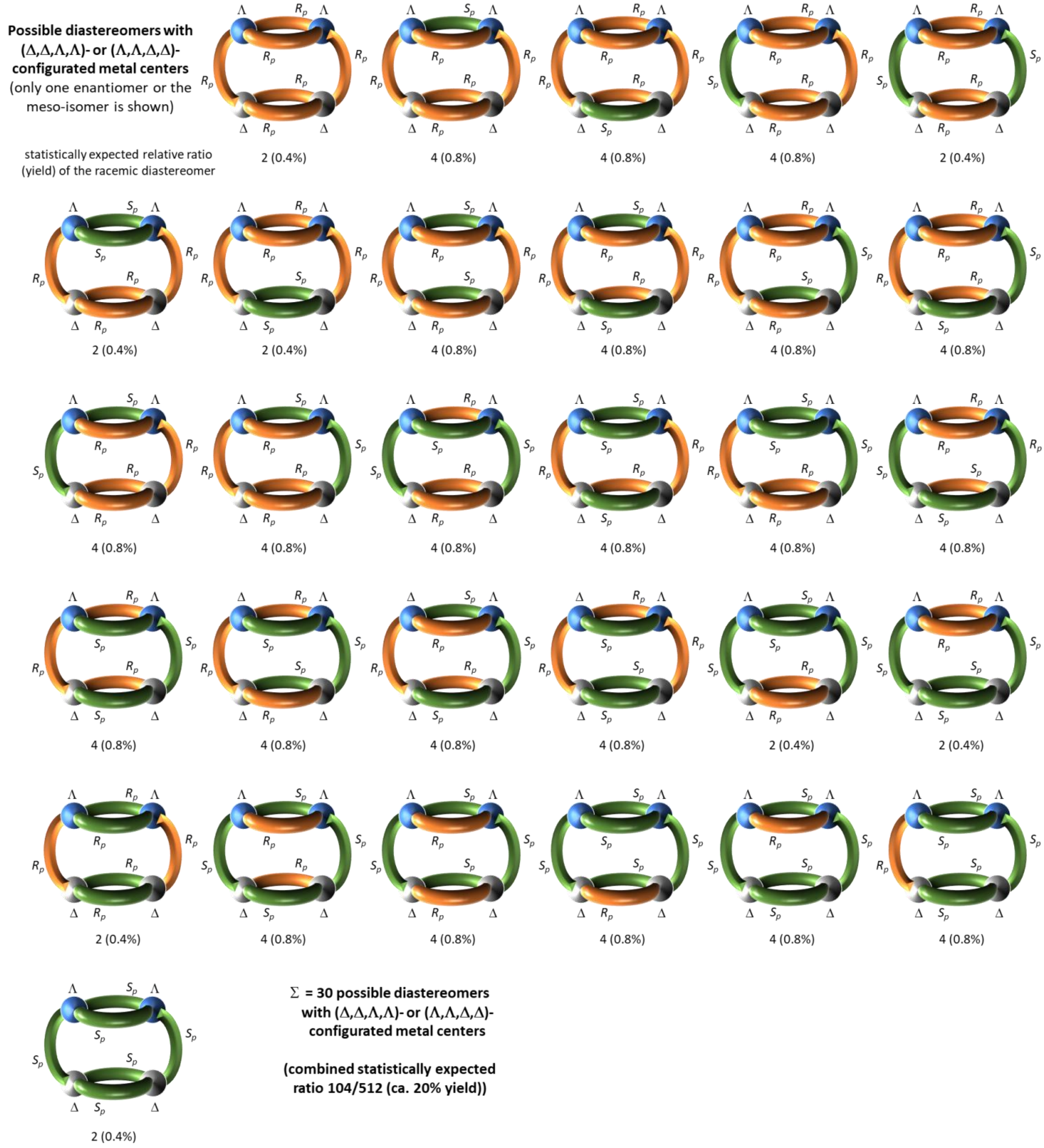
Possible diastereomers with
(Δ,Δ,Λ,Λ)- or (Λ,Λ,Δ,Δ)-
configurated metal centers
(only one enantiomer or the
meso-isomer is shown)
statistically expected relative ratio
(yield) of the racemic diastereomer
2 (0.4%)
4 (0.8%)
4 (0.8%)
4 (0.8%)
2 (0.4%)
2 (0.4%)
2 (0.4%)
4 (0.8%)
4 (0.8%)
4 (0.8%)
4 (0.8%)
4 (0.8%)
4 (0.8%)
4 (0.8%)
4 (0.8%)
4 (0.8%)
4 (0.8%)
4 (0.8%)
4 (0.8%)
4 (0.8%)
4 (0.8%)
2 (0.4%)
2 (0.4%)
2 (0.4%)
4 (0.8%)
4 (0.8%)
4 (0.8%)
4 (0.8%)
4 (0.8%)
2 (0.4%)
Σ = 30 possible diastereomers
with (Δ,Δ,Λ,Λ)- or (Λ,Λ,Δ,Δ)-
configurated metal centers
(combined statistically expected
ratio 104/512 (ca. 20% yield))

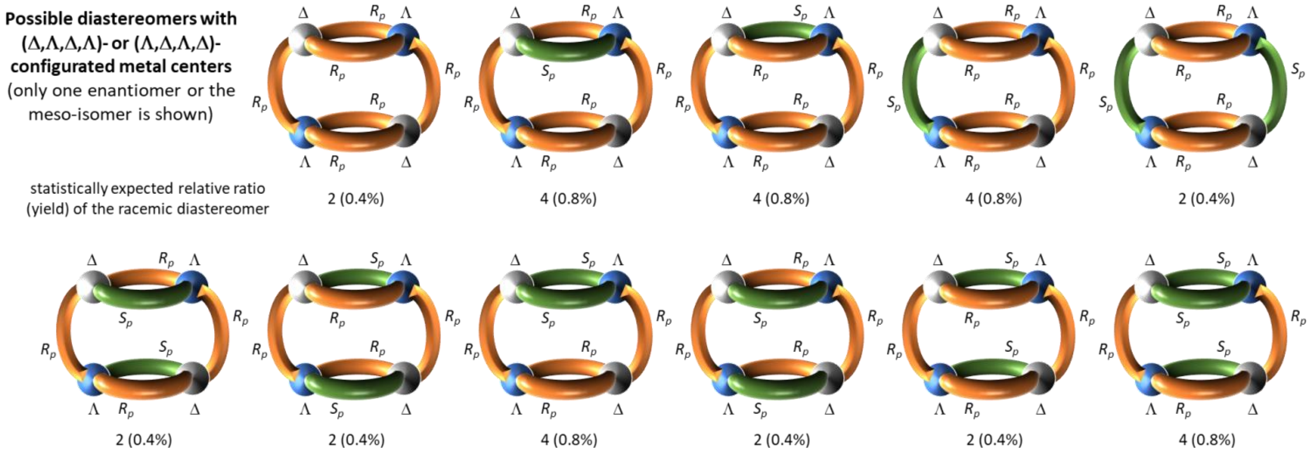
Possible diastereomers with
(Δ,Λ,Δ,Λ)- or (Λ,Δ,Λ,Δ)-
configurated metal centers
(only one enantiomer or the
meso-isomer is shown)
statistically expected relative ratio
(yield) of the racemic diastereomer
2 (0.4%)
4 (0.8%)
4 (0.8%)
4 (0.8%)
2 (0.4%)
2 (0.4%)
2 (0.4%)
4 (0.8%)
2 (0.4%)
2 (0.4%)
4 (0.8%)

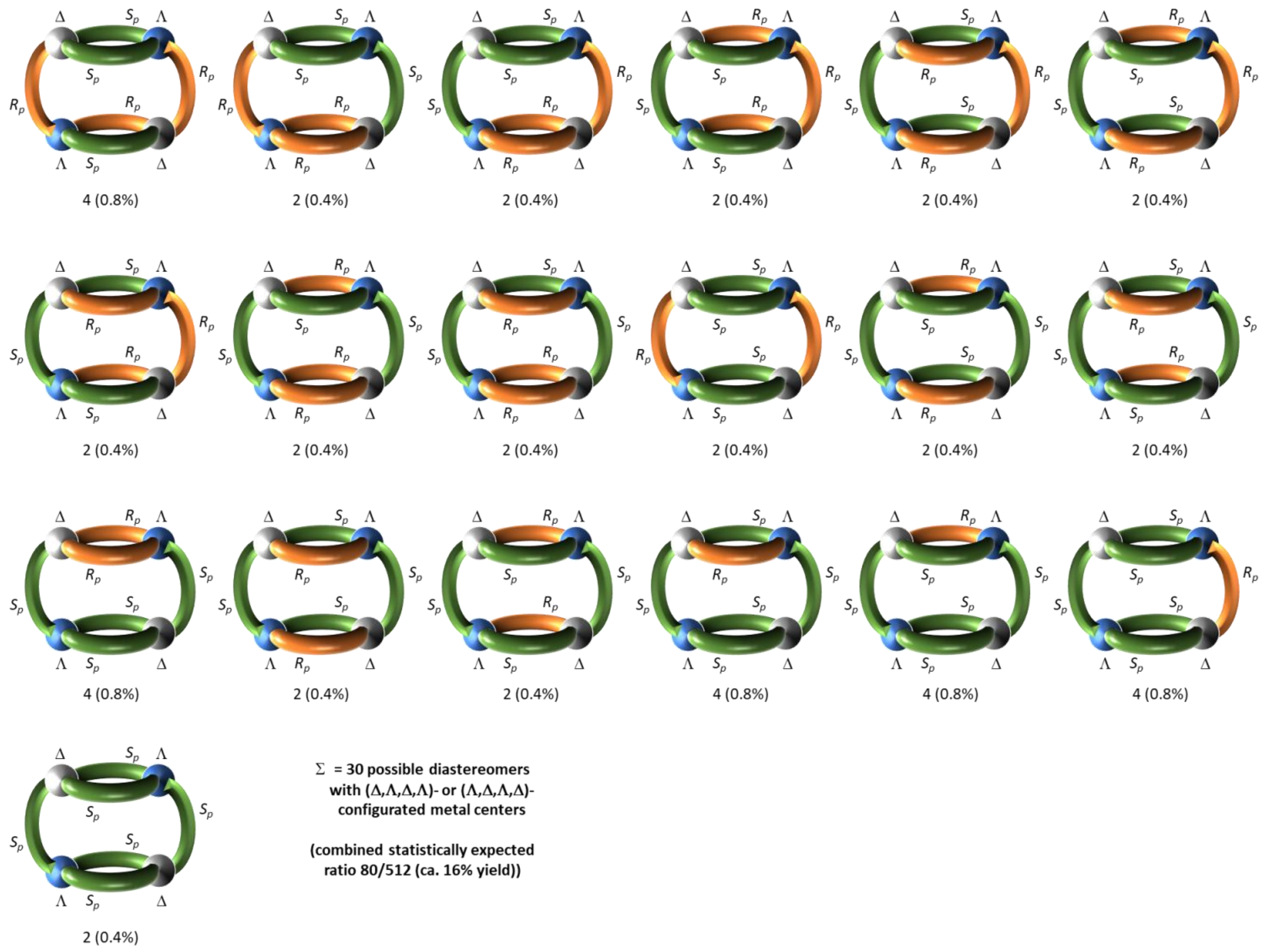
4 (0.8%)
2 (0.4%)
2 (0.4%)
2 (0.4%)
2 (0.4%)
2 (0.4%)
2 (0.4%)
2 (0.4%)
2 (0.4%)
2 (0.4%)
2 (0.4%)
2 (0.4%)
4 (0.8%)
2 (0.4%)
2 (0.4%)
4 (0.8%)
4 (0.8%)
4 (0.8%)
2 (0.4%)
Σ = 30 possible diastereomers with (Δ,Λ,Δ,Λ)- or (Λ,Δ,Λ,Δ)-configurated metal centers
(combined statistically expected ratio 80/512 (ca. 16% yield))

X-ray crystallography

X-Ray crystal structure experimental details for (S_p)-**1**, enantiomerically pure (Δ,Δ,Δ,Δ)-[Fe_4\{(R_p)-**L**\}$_6$]$(OTf)_8$ and (Λ,Λ,Λ,Λ)-[Fe_4\{(S_p)-**L**\}$_6$]$(OTf)_8$ and the racemate of (Δ,Δ,Δ,Δ)-[Fe_4\{(R_p)-**L**\}$_6$]$(OTf)_8$ and (Λ,Λ,Λ,Λ)-[Fe_4\{(S_p)-**L**\}$_6$]$(OTf)_8$ are given below. The single-crystal X-ray data for all structures were measured using a dual-source Rigaku SuperNova diffractometer equipped with an Atlas detector using mirror-monochromated Cu-Kα (λ = 1.54184 Å) radiation.[4] The data collection and reduction were performed using the program *CrysAlisPro*[1] and Gaussian face index absorption correction method[1] was applied. All structures were solved using direct methods (SHELXS)[5] and refined by full-matrix least squares based on F^2 using the *OLEX2* software[6] that utilizes the SHELXL-2015 module.[5]

Crystal structure refinement details: In Fe(II)-complexes, not all triflate anions to balance the metal ion positive charge could be found using electron Fourier difference map. This is due to weakly diffracting power of the crystals and severely disordered solvent molecules or electron residuals. Attempts to squeeze triflate anions and acetonitrile solvent molecules leaves Q-peaks ranging between 3.0 and 7.0 e/Å^3 in the final refinement. Therefore, restrains/constrains are applied to give roughly tetrahedral geometry for $-SO_3$ and $-CF_3$ groups in the triflate counterions.

Crystal data for (S_p)-**1**: CCDC-1919439, $C_{29}H_{30}N_2O$, M = 422.55, colourless plate, 0.115x0.108x0.08 mm^3, orthorhombic, space group $P2_12_12_1$, a = 8.06940(10) Å, b = 12.71500(10) Å, c = 22.9022(2) Å, α = 90°, β = 90°, γ = 90°, V = 2349.82(4) Å^3, Z = 4, Dc = 1.194 g/cm^3, F000 = 904, μ= 0.558 mm^{-1}, T = 120.0(1) K, $θ_{max}$= 76.908°, 42957 total reflections, 4777 with Io > 2σ(Io), R_{int} = 0.0322, 4927 data, 291 parameters, 0 restraints, GooF = 1.041, R = 0.0297 and wR = 0.0791 [Io > 2σ(Io)], R = 0.0309 and wR = 0.0801 (all reflections), 0.174<dΔρ<-0.140e/Å^3, Flack = -0.04(8).

Crystal data for (Δ,Δ,Δ,Δ)-[Fe_4\{(R_p)-**L**\}$_6$]$(OTf)_8$: CCDC-1919440, $C_{247}H_{195}F_{15}Fe_4N_{25}O_{15}S_5$, M = 4421.97, purple block, 0.222x0.123x0.116 mm^3, orthorhombic, space group $P2_12_12_1$, a = 28.0369(12) Å, b = 35.6387(7) Å, c = 41.0905(10) Å, α = 90°, β = 90°, γ = 90°, V = 41058(2) Å^3, Z = 4, Dc = 0.715 g/cm^3, F000 = 9164, μ= 1.710 mm^{-1}, T = 128(12) K, $θ_{max}$ = 56.021°, 270876 total reflections, 26113 with Io > 2σ(Io), R_{int} = 0.1085, 52293 data, 2555 parameters, 90 restraints, GooF = 1.007, R = 0.0883 and wR= 0.2543 [Io > 2σ(Io)], R = 0.1520 and wR= 0.3016 (all reflections), 0.558<dΔρ<-0.482 e/Å^3, Flack = 0.045(5).

Crystal data for (Λ,Λ,Λ,Λ)-[Fe_4\{(S_p)-**L**\}$_6$]$(OTf)_8$: CCDC-1919441, $C_{243}H_{195}F_3Fe_4N_{25}O_3S$, M = 3825.69, purple block, 0.11x0.09x0.09 mm^3, orthorhombic, space group $P2_12_12_1$, a = 28.6877(5) Å, b = 35.6914(6) Å, c = 41.3557(7) Å, α = 90°, β = 90°, γ = 90°, V = 42344.3(13) Å^3, Z = 4, Dc = 0.600 g/cm^3, F000 = 7996, μ= 1.376 mm^{-1}, T = 120.0(1) K, $θ_{max}$ = 55.731°, 503946 total reflections, 27281 with Io > 2σ(Io), R_{int} = 0.1206, 54068 data, 2033 parameters, 96 restraints, GooF = 1.033, R = 0.0885 and wR= 0.2642 [Io > 2σ(Io)], R = 0.1539 and wR= 0.3125 (all reflections), 0.560<dΔρ<-0.483 e/Å^3, Flack = 0.061(5).

Crystal data for the racemic mixture of (Δ,Δ,Δ,Δ)-[Fe_4\{(R_p)-**L**\}$_6$]$(OTf)_8$ and (Λ,Λ,Λ,Λ)-[Fe_4\{(S_p)-**L**\}$_6$]$(OTf)_8$: CCDC-1919442, $C_{244}H_{192}F_{12}Fe_4N_{24}O_{12}S_4$, M = 4231.84, purple block, 0.147x0.145x0.041 mm^3, tetragonal, space group $I4_1/acd$, a = 24.8852(3) Å, b = 24.8852(3) Å, c = 95.426(2) Å, α = 90°, β = 90°, γ = 90°, V = 59095(2) Å^3, Z = 8, Dc = 0.951 g/cm^3, F000 = 17568, μ= 2.270 mm^{-1}, T = 120.0(1) K, $θ_{max}$ = 38.216°, 23669 total reflections, 2747 with Io > 2σ(Io), R_{int} = 0.0502, 3912 data, 289 parameters, 50 restraints, GooF = 2.091, R = 0.1795 and wR= 0.4825 [Io > 2σ(Io)], R = 0.2082 and wR= 0.5100 (all reflections), 0.736<dΔρ<-0.880 e/Å^3.

[4] Rigaku Oxford Diffraction, 2017, *CrysAlisPro* Software system, version 38.46, Rigaku Corporation, Oxford, UK.

[5] a) G. M. Sheldrick, *Acta Crystallogr. Sect. C*, 2015, **71**, 3-8; b) G. M. Sheldrick, *Acta Crystallogr. Sect. A*, 2015, **71**, 3-8.

[6] O. V Dolomanov, L. J. Bourhis, R. J. Gildea, J. A. K. Howard and H. Puschmann, *J. Appl. Crystallogr*., 2009, **42**, 339-341.

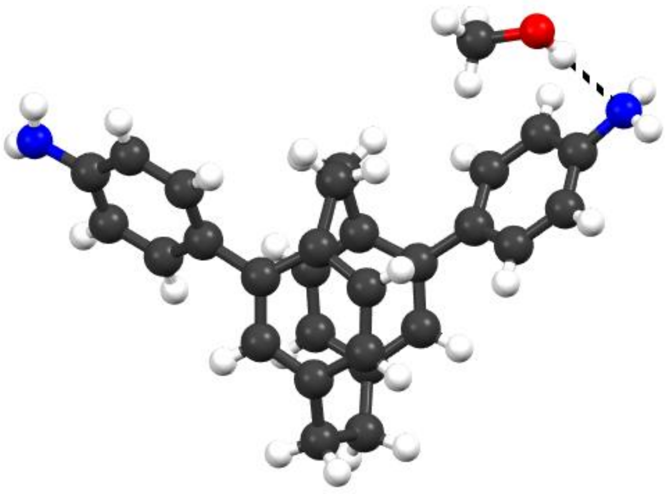

Figure S29. X-ray crystal structure of (S_p)-**1** in ball and stick model. Black broken lines represent hydrogen bonding interactions.

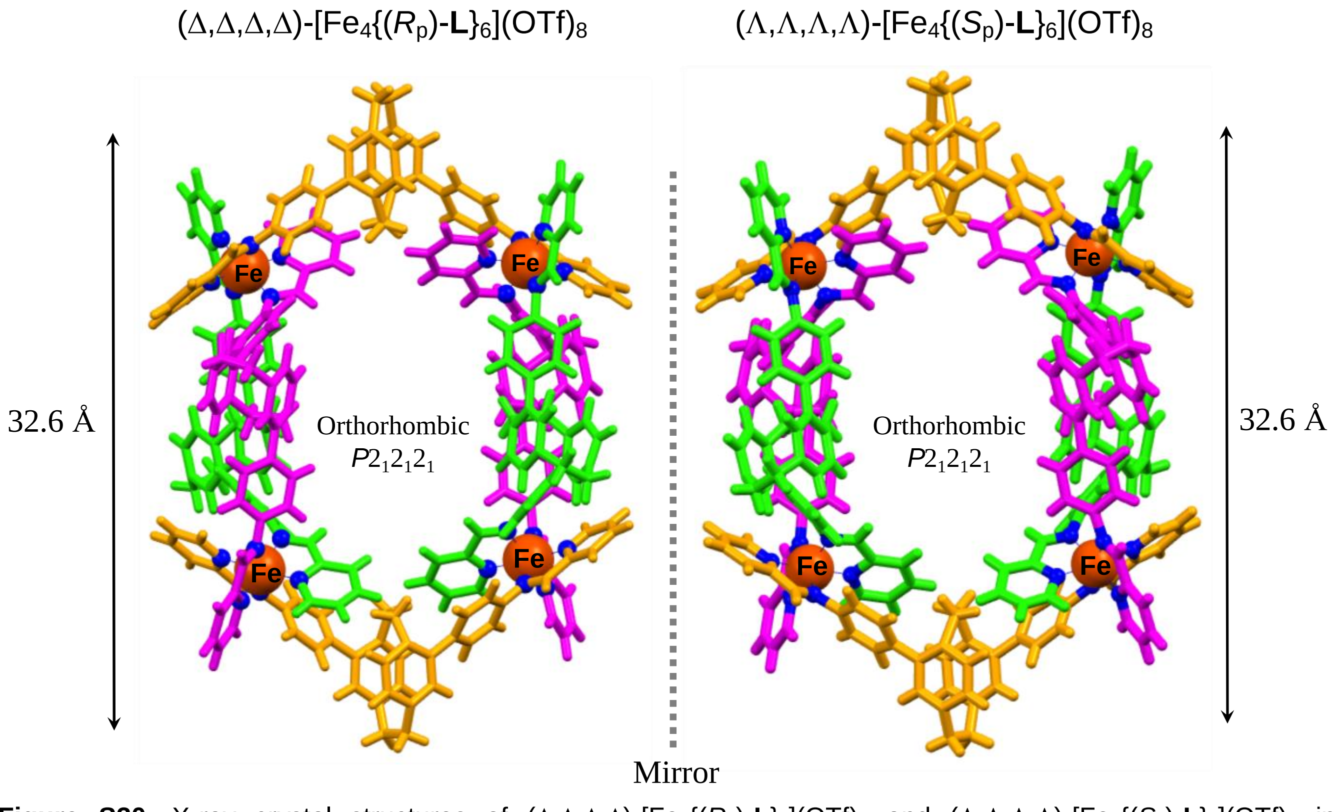

Figure S30. X-ray crystal structures of (Δ,Δ,Δ,Δ)-[Fe_4{(R_p)-**L**}$_6$]($OTf)_8$ and (Λ,Λ,Λ,Λ)-[Fe_4{(S_p)-**L**}$_6$]($OTf)_8$ in capped-stick model. Counterions were omitted for viewing clarity.

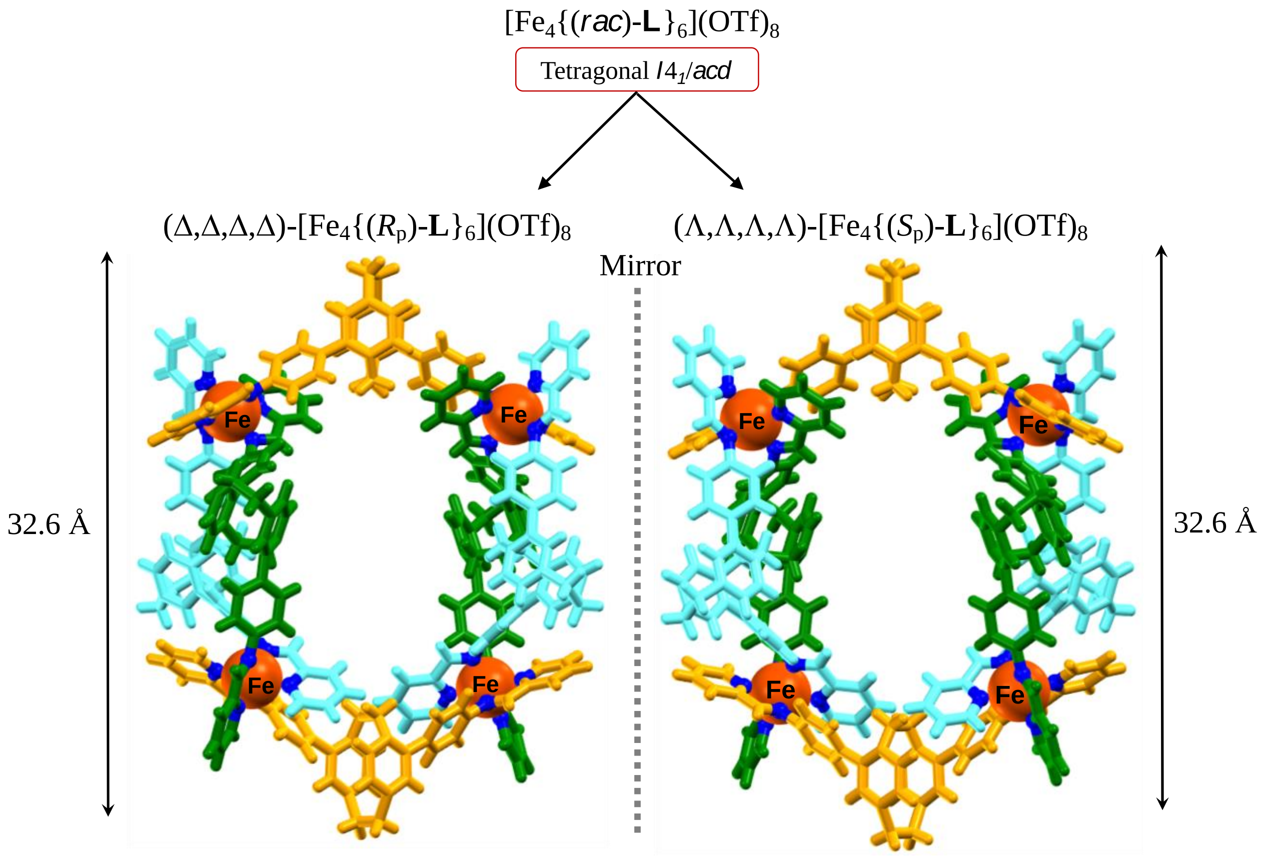

Figure S31. X-ray crystal structure of the racemic mixture of (Δ,Δ,Δ,Δ)-[Fe_4{(R_p)-**L**}$_6$]$(OTf)_8$ and (Λ,Λ,Λ,Λ)-[Fe_4{(S_p)-**L**}$_6$]$(OTf)_8$ depicted in isomeric forms using capped-stick model. Counterions were omitted for viewing clarity.
